# A non-intrusive global/local approach applied to phase-field modeling of brittle fracture

**DOI:** 10.1186/s40323-018-0105-8

**Published:** 2018-05-18

**Authors:** Tymofiy Gerasimov, Nima Noii, Olivier Allix, Laura De Lorenzis

**Affiliations:** 1 Institute of Applied Mechanics, Technische Universität Braunschweig, Pockelsstraße 3, 38106 Braunschweig, Germany; 2 LMT-Cachan, ENS Cachan, 61 Avenue du Président Wilson, 94235 Cachan Cedex, France

**Keywords:** Brittle fracture, Phase-field approach, Global/local formulation, Non-intrusive computations, Relaxation techniques, Convergence acceleration

## Abstract

This paper aims at investigating the adoption of non-intrusive global/local approaches while modeling fracture by means of the phase-field framework. A successful extension of the non-intrusive global/local approach to this setting would pave the way for a wide adoption of phase-field modeling of fracture, already well established in the research community, within legacy codes for industrial applications. Due to the extreme difference in stiffness between the global counterpart of the zone to be analized locally and its actual response when undergoing extensive cracking, the main foreseen issues are robustness, accuracy and efficiency of the fixed point iterative algorithm which is at the core of the method. These issues are tackled in this paper. We investigate the convergence performance when using the native global/local algorithm and show that the obtained results are identical to the reference phase-field solution. We also equip the global/local solution update procedure with relaxation/acceleration techniques such as Aitken’s $\Delta ^2$-method, the Symmetric Rank One and Broyden’s methods and show that the iterative convergence can be improved significantly. Results indicate that Aitken’s $\Delta ^2$-method is probably the most convenient choice for the implementation of the approach within legacy codes, as this method needs only tools already available for the so-called sub-modeling approach, a strategy routinely used in industrial contexts.

## Introduction

**Fig. 1:**
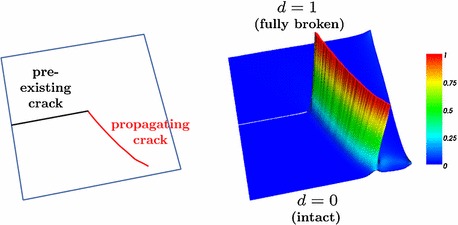
Phase-field description of fracture (sketchy): $d\in C(\Omega ,[0,1])$ is the crack phase-field

The variational approach to fracture by Francfort and Marigo [1] and the related regularized formulation of Bourdin et al. [2–5], commonly referred to as phase-field model of (brittle) fracture, is a widely accepted framework for modeling and computing fracture phenomena in elastic solids. The phase-field framework for modeling systems with sharp interfaces consists in incorporating a continuous field variable—the so-called order parameter—which differentiates between multiple physical phases within a given system through a smooth transition. In the context of fracture, such an order parameter (termed the *crack phase-field*) describes the smooth transition between the fully broken and intact material phases, thus approximating the sharp crack discontinuity, as sketched in Fig. 1. The evolution of this field as a result of the external loading conditions models the fracture process. The formulation is strongly non-linear and calls for the resolution of small length scales.

The use of phase-field approaches in the case of structures of industrial complexity has been the subject of limited investigations thus far and poses a number of challenges. In this paper, in order to move forward in this direction we advocate the use of non-intrusive global/local strategies initially proposed in [6]. When dealing with large structures, fracture phenomena most often occur in regions of limited extent only. Moreover, in the case of brittle fracture most of the structure behaves elastically. These features are particularly appealing for global/local approaches as they make it possible to first compute the global model elastically, and then determine the critical areas to be re-analyzed, while storing the factorization of the decomposition of the structural stiffness. The local models are then iteratively substituted within the unchanged global one, which has the advantage of avoiding the reconstruction of the mesh of the whole structure. In fact, this is the main motivation of non-intrusive global/local approaches: to avoid the modification of the finite element model used by engineers, the creation of a complex global model being by far the most time-consuming task, a task which is more and more externalized.

In the past decade, both phase-field and non-intrusive global/local approaches have been extended to deal with a growing number of situations of interest for engineers. The currently available phase-field formulations of brittle fracture encompass static and dynamic models. We mention the papers by Amor et al. [7], Miehe et al. [8, 9], Kuhn and Müller [10], Pham et al. [11], Borden et al. [12], Mesgarnejad et al. [13], Kuhn et al. [14], Ambati et al. [15], Wu et al. [16], where various formulations are developed and validated. Recently, the framework has been also extended to ductile (elasto-plastic) fracture [17–22], pressurized fracture in elastic and porous media [23, 24], fracture in films [25] and shells [26–28], and multi-field fracture [29–36]. Non-intrusive global/local approaches have also been applied to a quite large number of situations: the computation of the propagation of cracks in a sound model using the extended finite element method (XFEM) [37], the computation of assembly of plates introducing realistic non-linear 3D modeling of connectors [38], the extension to non-linear domain decomposition methods [39] and to explicit dynamics [40, 41] with an application to the prediction of delamination under impact using Abaqus [42]. Alternative strategies can be derived from the Partition of Unity Method [43, 44].

The phase-field simulation of fracture processes with legacy codes bears a number of advantages which fit perfectly within the framework of non-intrusive coupling approaches using pre-defined ‘fixed’ meshes. The most obvious advantage is the ability to track automatically a cracking process by the evolution of the smooth crack field on a ‘fixed’ mesh which, in the proposed procedure, is the mesh of the local model. This is a significant advantage over the discrete fracture description, whose numerical implementation requires explicit (in the classical finite element method, FEM) or implicit (within XFEM) handling of the discontinuities. The possibility to avoid the tedious task of tracking complicated crack surfaces in 3D significantly simplifies the implementation. The second advantage is the ability to simulate complicated processes, including crack initiation (also in the absence of a crack tip singularity), propagation, coalescence and branching without the need for additional ad-hoc criteria and with very few parameters to be identified. This feature is particularly attractive for industrial applications, as it minimizes the need for time-consuming and expensive calibration tests.

Due to the extreme difference in stiffness between the global counterpart of the zone to be re-analyzed locally and its actual response when undergoing extensive cracking, the foreseen fundamental issues associated with the use of the global/local strategy in combination with phase-field fracture modeling are robustness, accuracy and efficiency of the fixed point iterative algorithm which is at the core of the method. Also, the finite element treatment of the phase-field formulation of brittle fracture is known to be computationally demanding, mainly due to the non-convexity of the energy functional to be minimized with respect to both arguments (the displacement and the phase field) simultaneously [45–47]. As a result, the so-called monolithic approach manifests major iterative convergence issues of the Newton–Raphson procedure. A new line-search scheme [46] and modified Newton methods [47] have been recently proposed to tackle this problem. Alternatively, staggered (also termed partitioned, or alternate minimization) solution scheme is widely used. This is based on decoupling of the strongly non-linear weak formulation into a system and then iterating between the equations [2–5, 7–9, 11–13, 15, 16]. The staggered scheme is proved to be robust, but typically has a very slow convergence behavior of the iterative solution process, see e.g. [15, 46, 48]. In view of the above, a central question that arises when combining non-intrusive global/local approaches with phase-field modeling of fracture is how additional global/local iterations affect and possibly deteriorate the highly sensitive iterative behavior of the staggered scheme used to solve the phase-field equations. In this paper, we make a first attempt to address these questions.

The paper is organized as follows. In “The phase-field approach to brittle fracture” section, we outline the main concepts of phase-field modeling of brittle fracture and illustrate the specific formulation used in the present paper. “Global/local approach in a non-intrusive setting” section introduces the non-intrusive global/local approach for the solution of the reference phase-field model considered in “The phase-field approach to brittle fracture” section. This is done in several steps. We start by illustrating an intrusive global/local scheme through a domain decomposition formulation in a variational setting well adapted to the phase field formulation. Several options are considered, including the so-called primal, dual and localized Lagrange multipliers based versions. This domain decomposition framework is used afterwards to define some convergence indicators in terms both of incompatibility of the reaction forces and of displacement jumps at the interface between the unchanged global model and the re-analyzed local one. The motivation here is to see which indicator or combination of indicators are the most suited to an appropriate estimation of the quality of the global/local iteration results with respect to the phase-field determination. The third version is then extended to the global/local setting, for which a non-intrusive computational procedure is devised. The numerical results which illustrate the performance of the proposed non-intrusive global/local approach as well as their qualitative and quantitative comparison with the reference solution are reported in “Results and discussion” section. Therein, we also outline and apply three relaxation/acceleration techniques, which are incorporated into the global/local iterative procedure and aim at improving its efficiency. Conclusions and outlook finalize the paper.

## The phase-field approach to brittle fracture

In this section, we consider a mechanical system undergoing a brittle fracture process modeled with the phase-field formulation, and term this the *reference* problem. For this problem, we develop in “Global/local approach in a non-intrusive setting” section a global/local formulation, which is dissected numerically in “Results and discussion” section.

**Fig. 2:**
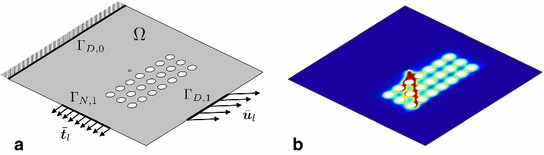
**a** Sketch of geometry and loading setup; **b** the computed crack phase-field evolution

Let $\Omega \subset \mathbb {R}^m$, $m=2$ or 3 be an open and bounded domain representing the configuration of a *m*-dimensional linear elastic body, and let $\Gamma _{D,0},\Gamma _{D,1}$ and $\Gamma _{N,1}$ be the (non-overlapping) portions of the boundary $\partial \Omega$ of $\Omega$ on which homogeneous Dirichlet, non-homogeneous Dirichlet and Neumann boundary conditions are prescribed, respectively. In the following, we consider a quasi-static loading process with the discrete pseudo-time step parameter $l=0,1,...$, such that the displacement $\bar{\varvec{u}}_l$ and traction $\bar{\varvec{t}}_l$ loading data are prescribed on the corresponding parts of the boundary, see Fig. 2a.

For the mechanical system at hand, the phase-field formulation of brittle fracture [5] in an *incremental variational setting* relies on the following energy functional

1 $$\begin{aligned} \mathcal {E}(\varvec{u},d) = \int _\Omega W(\varvec{\varepsilon }(\varvec{u}),d) \, \mathrm {d}{} \mathbf x -\int _{\Gamma _{N,1} } \bar{\varvec{t}}_l \cdot {\varvec{u}}\,\mathrm {d}s. \end{aligned}$$

with

2 $$\begin{aligned} W(\varvec{\varepsilon }(\varvec{u}),d):= (1-d)^2\Psi ^+(\varvec{\varepsilon }(\varvec{u}))+\Psi ^-(\varvec{\varepsilon }(\varvec{u})) + \frac{G_c}{2}\left( \frac{d^2}{\ell }+\ell |\nabla d|^2\right) , \end{aligned}$$

and the related minimization problem at each $l\ge 0$. In the above, the displacement field $\varvec{u}:\Omega \rightarrow \mathbb {R}^m$ and the crack phase-field $d:\Omega \rightarrow [0,1]$ are the arguments of $\mathcal {E}$. As already mentioned, the limiting values of *d*, namely, $d=0$ and $d=1$ represent the undamaged and fully broken material phases. Furthermore, $\Psi ^+$ and $\Psi ^-$ are the so-called ‘tensile’ and ‘compressive’ parts of an additive decomposition of the elastic strain energy density function $\Psi (\varvec{\varepsilon }):=\frac{1}{2}\varvec{\varepsilon }:\mathbb {C}:\varvec{\varepsilon }=\frac{1}{2}\lambda \mathrm {tr}^2(\varvec{\varepsilon })+\mu \mathrm {tr}(\varvec{\varepsilon }\cdot \varvec{\varepsilon })$, where, in turn, $\varvec{\varepsilon }$ is the second-order infinitesimal strain tensor, $\mathbb {C}$ is the fourth-order elasticity tensor, and $\lambda$ and $\mu$ are the Lamé constants. The decomposition of $\Psi$ into $\Psi ^+$ and $\Psi ^-$ is required in order to distinguish between fracture behavior in tension and compression, more precisely, to avoid crack growth and crack faces interpenetration in compression. Here we use the spectral-based split, proposed in [8, 9]:

3 $$\begin{aligned} \Psi ^\pm (\varvec{\varepsilon }):= \frac{1}{2}\lambda \langle \mathrm {tr}(\varvec{\varepsilon }) \rangle _{\pm }^2 + \mu \mathrm {tr}(\varvec{\varepsilon }_\pm \cdot \varvec{\varepsilon }_\pm ), \end{aligned}$$

where $\langle a\rangle _{\pm }:=\frac{1}{2}(a\pm |a|)$ and $\varvec{\varepsilon }_{\pm }:=\sum _{I=1}^3\langle \varepsilon _I\rangle _{\pm }\varvec{n}_I\otimes \varvec{n}_I$ with $\{\varepsilon _I\}_{I=1}^3$ and $\{\varvec{n}_I\}_{I=1}^3$ as the principal strains and principal strain directions, respectively. Finally, $G_c$ is the material fracture toughness, and $0<\ell \ll \mathrm {diam}(\Omega )$ is the regularization parameter that controls the width of the transition zone of *d* between the two material states.

With $\mathcal {E}$ defined by (1), the state of the system at a given loading step $l\ge 0$ is then represented by the solution of

4 $$\begin{aligned} \mathrm {arg\,min} \{ \mathcal {E}(\varvec{u},d): \; {\varvec{u}}\in \mathbf V _{\bar{\varvec{u}}_l}, d\in \mathcal {D}_{d_{l-1}} \}, \end{aligned}$$

where

$$\begin{aligned} \mathbf V _{\bar{\varvec{u}}_l}:=\{ \varvec{u}\in \mathbf H ^1(\Omega ): \; \varvec{u}=\varvec{0} \; \mathrm {on} \; \Gamma _{D,0}, \; \varvec{u}=\bar{\varvec{u}}_l\; \mathrm {on} \; \Gamma _{D,1} \} \end{aligned}$$

is the kinematically admissible displacement space with $\mathbf H ^1(\Omega ):=[H^1(\Omega )]^m$ and $H^1$ denoting the usual Sobolev space, and

$$\begin{aligned} \mathcal {D}_{d_{l-1}}:=\{ d\in H^1(\Omega ,[0,1]): \; d_{l-1}\le d \} \end{aligned}$$

is the admissible space for *d* with $d_{l-1}$ being known from the previous step. The condition $d_{l-1}\le d$ is used to enforce the irreversibility of the crack phase-field evolution. Figure 2b depicts an example of phase-field pattern resulting from (4).

Note that due to the $d_{l-1}\le d$ requirement, problem (4) is a constrained minimization problem and its necessary optimality condition which enables computing the solution $(\varvec{u},d)\in \mathbf V _{\bar{\varvec{u}}_l}\times \mathcal {D}_{d_{l-1}}$ is a variational inequality. Its partitioned form reads as

5 $$\begin{aligned} \left\{ \begin{array}{ll} {\mathcal {E}}_{\varvec{u}}(\varvec{u},d;\varvec{v})=0, \qquad \quad \, \forall \; \varvec{v}\in \mathbf V _0, \\ {\mathcal {E}}_d(\varvec{u},d;w-d)\ge 0, \quad \forall \;w\in \mathcal {D}_{d_{l-1}}, \end{array} \right. \end{aligned}$$

see e.g. [11, 48], where ${\mathcal {E}}_{\varvec{u}}$ and ${\mathcal {E}}_d$ are the directional derivatives of the energy functional with respect to $\varvec{u}$ and *d*, respectively. It is

6 $$\begin{aligned} {\mathcal {E}}_{\varvec{u}}(\varvec{u},d;\varvec{v}):= & {} \int _\Omega \left[ (1-d)^2\frac{\partial \Psi ^+}{\partial \varvec{\varepsilon }}(\varvec{\varepsilon }(\varvec{u}))+\frac{\partial \Psi ^-}{\partial \varvec{\varepsilon }}(\varvec{\varepsilon }(\varvec{u}))\right] :\varvec{\varepsilon }(\varvec{v})\,\mathrm {d}{} \mathbf x -\int _{\Gamma _{N,1} } \bar{\varvec{t}}_l \cdot \varvec{v}\,\mathrm {d}s, \end{aligned}$$

7 $$\begin{aligned} {\mathcal {E}}_d(\varvec{u},d;w):= & {} \int _\Omega \left[ -\,2(1-d)\Psi ^+(\varvec{\varepsilon }(\varvec{u}))w +G_c\left( \frac{1}{\ell }dw + \ell \nabla {d}\cdot \nabla {w} \right) \right] \,\mathrm {d}{} \mathbf x . \end{aligned}$$

The displacement test space in (5) is defined as $\mathbf V _0:=\{ \varvec{v}\in \mathbf H ^1(\Omega ): \varvec{v}=\varvec{0} \; \mathrm {on} \; \Gamma _{D,0}\cup \Gamma _{D,1} \}$.

In (6), the components $\frac{\partial \Psi ^\pm }{\partial \varvec{\varepsilon }}$ are the corresponding ‘tensile’ and ‘compressive’ stresses, which are strongly non-linear in $\varvec{\varepsilon }$. In the case of the spectral-based split in (3), we obtain

8 $$\begin{aligned} \varvec{\sigma }^\pm (\varvec{\varepsilon }):= \frac{\partial \Psi ^\pm }{\partial \varvec{\varepsilon }}(\varvec{\varepsilon }) = \lambda \langle \mathrm {tr}(\varvec{\varepsilon }) \rangle _{\pm }\varvec{I} + 2\mu \varvec{\varepsilon }_\pm . \end{aligned}$$

The related counterparts of the standard fourth-order elasticity tensor $\mathbb {C}$ read in this case

9 $$\begin{aligned} \mathbb {C}^\pm (\varvec{\varepsilon }):= \frac{\partial \varvec{\sigma }^\pm }{\partial \varvec{\varepsilon }}(\varvec{\varepsilon }) = \lambda H^\pm (\mathrm {tr}(\varvec{\varepsilon })) \mathbb {J} + 2\mu \mathbb {P}^\pm (\varvec{\varepsilon }), \end{aligned}$$

where $H^+$ is the standard Heaviside function and $H^-:=1-H^+$, $\mathbb {J}$ is the fourth-order symmetric identity tensor, whereas $\mathbb {P}^\pm$ are the fourth-order tensors obtained by differentiation of $\varvec{\varepsilon }_\pm$ with respect to $\varvec{\varepsilon }$.

Stemming from the irreversibility constraint $d_{l-1}\le d$ the variational inequality ${\mathcal {E}}_d\ge 0$ in (5) requires special solution algorithms, see e.g. [49, 50]. Here, the irreversibility of *d* is enforced ‘indirectly’ via the notion of a *history* variable, as proposed in Miehe et al. [9]. The idea is that the tensile energy $\Psi ^+$ can be viewed as the driving force of the phase-field evolution. Hence, the maximal $\Psi ^+$ accumulated within the loading history and denoted as $\mathcal {H}_l(\mathbf x ):=\mathrm {max}_{\forall l}\Psi ^+(\varvec{\varepsilon }(\varvec{u}))$ can be used to prevent a decrease of the phase-field. $\mathcal {H}_l$ substitutes the corresponding $\Psi ^+$ term in the original $\mathcal {E}_d$, thus yielding

10 $$\begin{aligned} {\mathcal {E}}^*_d(\varvec{u},d;w):=\int _\Omega \left[ -2(1-d)\mathcal {H}_l w +G_c\left( \frac{1}{\ell }d w + \ell \nabla {d}\cdot \nabla {w} \right) \right] \,\mathrm {d}{} \mathbf x \end{aligned}$$

and the system for computing the solution $(\varvec{u},d)\in \mathbf V _{\bar{\varvec{u}}_l}\times H^1(\Omega )$ is

11 $$\begin{aligned} \left\{ \begin{array}{ll} {\mathcal {E}}_{\varvec{u}}(\varvec{u},d;\varvec{v})=0, \quad \forall \; \varvec{v}\in \mathbf V _0, \\ {\mathcal {E}}^*_d(\varvec{u},d;w)=0, \quad \forall \; w\in H^1(\Omega ), \end{array} \right. \end{aligned}$$

where ${\mathcal {E}}_{\varvec{u}}$ is given by (6). We obtain in (11) an equality and unconstrained spaces for *d* and *w*.

The staggered solution algorithm for the system in (11) implies alternately fixing $\varvec{u}$ and *d*, and solving the corresponding equations until convergence. The algorithm is sketched in Table 1.

**Table 1:** Staggered iterative solution process for (11) at a fixed loading step *l*

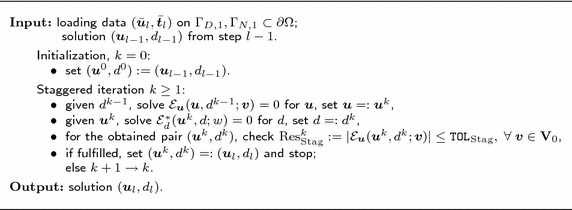	

Note that the equation ${\mathcal {E}}_{\varvec{u}}=0$ in Table 1 is strongly non-linear due to the non-linearity of $\varvec{\sigma }(\varvec{u},d):=(1-d)^2\frac{\partial \Psi ^+}{\partial \varvec{\varepsilon }}(\varvec{\varepsilon }(\varvec{u}))+\frac{\partial \Psi ^-}{\partial \varvec{\varepsilon }}(\varvec{\varepsilon }(\varvec{u}))$, see equation (8). Therefore, at every staggered iteration $k\ge 1$ with given $d^{k-1}$, a Newton–Raphson procedure is needed to compute $\varvec{u}^k$, with e.g. $\varvec{u}^{k-1}$ being taken as the initial guess, and $\texttt {TOL}_\mathrm {NR}$ as a user-defined tolerance. Owing to the ‘nested in’ nature of the Newton–Raphson process, it has to be $\texttt {TOL}_\mathrm {NR}<\texttt {TOL}_\mathrm {Stag}$. In the presented numerical examples we take $\texttt {TOL}_\mathrm {NR}:=10^{-8}<\texttt {TOL}_\mathrm {Stag}:=10^{-5}$.

## Global/local approach in a non-intrusive setting

The starting point towards *a non-intrusive global/local approach* to the phase-field problem (4) with $\mathcal {E}$ defined by (1) is a standard non-overlapping domain decomposition procedure applied to $\mathcal {E}$. The resulting formulation is then extended to a global/local one in the spirit of [39, 51], for which the non-intrusive computational scheme is devised.

### Domain decomposition formulation

**Fig. 3:**
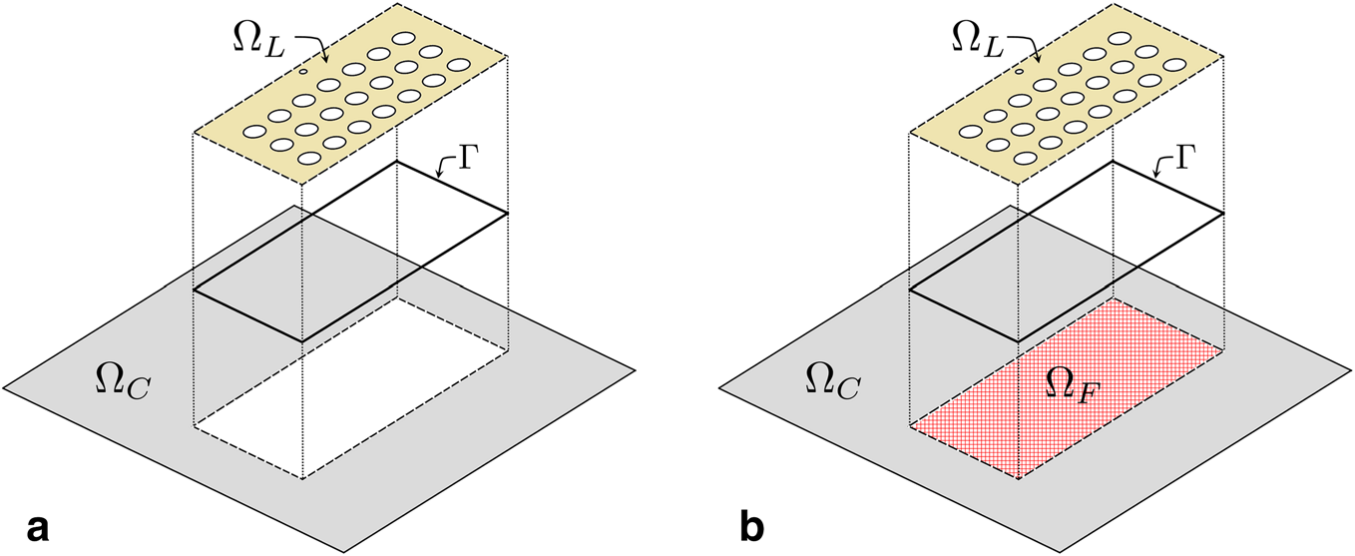
Domain decomposition procedure: **a** the classic one, when $\Omega$ is decomposed into local and complementary sub-domains $\Omega _L$ and $\Omega _C$, respectively, which do not overlap and are coupled by the interface $\Gamma$; **b** its extension to the global/local setting, where a fictitious domain $\Omega _F$ is introduced to form the so-called global domain $\Omega _G:=\Omega _C\cup \Gamma \cup \Omega _F$

Let $\Omega _L$ be an open sub-domain of $\Omega$, where cracking (in a general setting: a strong localization effect due to non-linearity) is expected to take place, and let $\Omega _C\subset \Omega$ be its open complement ($\Omega _C:=\Omega /\overline{\Omega _L}$), where the material remains intact and elastic (in a general setting: non-linearity is negligible). In the following, the subscripts *L* and *C* always stand for local and complementary, respectively. It is typical to assume that $\Omega _L$ represents a reasonably small ‘fraction’ of $\Omega$ such that $|\Omega _L|\ll |\Omega _C|$. Let also $\Gamma \subset \Omega$ be the interface between $\Omega _L$ and $\Omega _C$, a set with one dimension less than the dimension of $\Omega$, such that $\Omega _L\cup \Gamma \cup \Omega _C\equiv \Omega$. With an application to the problem sketched in Fig. 2, this domain decomposition idea is presented in Fig. 3a.

We now introduce two functions on $\Omega _L$ and $\Omega _C$, namely, $\varvec{u}_L\in \mathbf H ^1(\Omega _L)$ and $\varvec{u}_C\in \{ \mathbf H ^1(\Omega _C):\varvec{u}_C=\varvec{0} \; \mathrm {on} \; \Gamma _{D,0}, \; \varvec{u}_C=\bar{\varvec{u}}_l\; \mathrm {on} \; \Gamma _{D,1} \}$ such that

12 $$\begin{aligned} \varvec{u}_L\overset{!}{=}\varvec{u}_C \quad \text{ for } \; \mathbf x \in \Gamma , \end{aligned}$$

and assume that the displacement $\varvec{u}\in \mathbf V _{\bar{\varvec{u}}_l}$ stemming from the solution of problem (4), can be represented as

13 $$\begin{aligned} \varvec{u} =\left\{ \begin{array}{ll} \varvec{u}_L, &{} \quad \text { for }{} \mathbf x \in \Omega _L, \\ \varvec{u}_C, &{} \quad \text { for }{} \mathbf x \in \Omega _C. \end{array} \right. \end{aligned}$$

We furthermore introduce a function $d_L:\Omega _L\rightarrow [0,1]$ such that the phase-field $d\in \mathcal {D}_{d_{l-1}}$ stemming from the solution of (4) has the representation

14 $$\begin{aligned} d =\left\{ \begin{array}{ll} d_L, &{} \quad \text { for }{} \mathbf x \in \Omega _L, \\ 0, &{} \quad \text { for }{} \mathbf x \in \Omega _C. \end{array} \right. \end{aligned}$$

Using (13) and (14) in the function *W* in (2), we arrive at the energy functionals in the corresponding sub-domains, namely,

15 $$\begin{aligned} {\mathcal {E}}_1(\varvec{u}_C): = \int _{\Omega _C} W(\varvec{\varepsilon }(\varvec{u}_C),0) \,\mathrm {d}{} \mathbf x - \int _{\Gamma _{N,1} } \bar{\varvec{t}}_l \cdot \varvec{u}_C \,\mathrm {d}s, \end{aligned}$$

and

16 $$\begin{aligned} {\mathcal {E}}_2(\varvec{u}_L,d_L): =\int _{\Omega _L} W(\varvec{\varepsilon }(\varvec{u}_L),d_L) \,\mathrm {d}{} \mathbf x , \end{aligned}$$

such that, also owing to (12), it holds

17 $$\begin{aligned} {\mathcal {E}}(\varvec{u},d) \equiv \widehat{\mathcal {E}}(\varvec{u}_C,\varvec{u}_L,d_L) := {\mathcal {E}}_1(\varvec{u}_C)+{\mathcal {E}}_2(\varvec{u}_L,d_L), \end{aligned}$$

where, to recall, ${\mathcal {E}}$ is the original reference functional (1). As a result, the domain decomposition variational formulation, which is *equivalent* to reference formulation (4), reads

18 $$\begin{aligned} \underset{ \varvec{u}_C, \varvec{u}_L, d_L}{ \mathrm {arg\,min} }\; \widehat{\mathcal {E}}(\varvec{u}_C,\varvec{u}_L,d_L). \end{aligned}$$

The advantage of ‘replacing’ (4) with (23) is that one of the two sub-problems stemming from (18), more precisely, the complementary one, will be linear: indeed, $W(\varvec{\varepsilon }(\varvec{u}),0)=\Psi (\varvec{\varepsilon }(\varvec{u}))$, thus yielding the standard linear stress-strain relation $\varvec{\sigma }(\varvec{u}):=\frac{\partial W}{\partial \varvec{\varepsilon }}(\varvec{\varepsilon }(\varvec{u}),0)=\mathbb {C}:\varvec{\varepsilon }(\varvec{u})$. And this, moreover, will take place in a ‘large portion’ of $\Omega$, since by assumption $|\Omega _L|\ll |\Omega _C|$.

Due to the strong displacement continuity requirement (12), formulation (18) is called *primal* in the literature, see e.g. [52]. This requirement may be too restrictive from the computational standpoint [53]. Relaxing, or rather neglecting (12), results in the appearance of the traction-like terms in the corresponding sub-domain energy functionals (15) and (16):

19 $$\begin{aligned} {\mathcal {E}}_1(\varvec{u}_C,\varvec{\lambda }_C): = \int _{\Omega _C} W(\varvec{\varepsilon }(\varvec{u}_C),0) \,\mathrm {d}{} \mathbf x - \int _\Gamma \varvec{\lambda }_C\cdot \varvec{u}_C \,\mathrm {d}s - \int _{\Gamma _{N,1} } \bar{\varvec{t}}_l \cdot \varvec{u}_C \,\mathrm {d}s, \end{aligned}$$

and

20 $$\begin{aligned} {\mathcal {E}}_2(\varvec{u}_L,d_L,\varvec{\lambda }_L): =\int _{\Omega _L} W(\varvec{\varepsilon }(\varvec{u}_L),d_L) \,\mathrm {d}{} \mathbf x - \int _\Gamma \varvec{\lambda }_L\cdot \varvec{u}_L \,\mathrm {d}s, \end{aligned}$$

with $\varvec{\lambda }_C,\varvec{\lambda }_L\in \mathbf L ^2(\Gamma )$ being the (unknown) Lagrange multipliers, which represent tractions. In this case, however, the ‘$\mathrm {argmin\,max}$’-problem being posed for

$$\begin{aligned} \widehat{\mathcal {E}}(\varvec{u}_C,\varvec{u}_L,d_L,\varvec{\lambda }_L,\varvec{\lambda }_C) := {\mathcal {E}}_1(\varvec{u}_C,\varvec{\lambda }_C)+{\mathcal {E}}_2(\varvec{u}_L,d_L,\varvec{\lambda }_L), \end{aligned}$$

is under-determined, since no relation is yet specified between $\varvec{u}_L$ and $\varvec{u}_C$, nor between $\varvec{\lambda }_L$ and $\varvec{\lambda }_C$.

Two standard ways to proceed with (19) and (20), and obtaining a variational formulation equivalent to the original one in (4) are as follows.

*Option 1* One imposes a strong continuity between $\varvec{\lambda }_C$ and $\varvec{\lambda }_L$ on $\Gamma$, by setting in ${\mathcal {E}}_1$ and ${\mathcal {E}}_2$

21 $$\begin{aligned} \varvec{\lambda }_C\overset{!}{=}-\varvec{\lambda }_L=:\varvec{\lambda }. \end{aligned}$$

Summing the obtained functionals leads to

22 $$\begin{aligned} \widehat{\mathcal {E}}(\varvec{u}_C,\varvec{u}_L,d_L,\varvec{\lambda }):= & {} \int _{\Omega _C} W(\varvec{\varepsilon }(\varvec{u}_C),0) \,\mathrm {d}{} \mathbf x +\int _{\Omega _L} W(\varvec{\varepsilon }(\varvec{u}_L),d_L) \,\mathrm {d}{} \mathbf x \nonumber \\&+\int _\Gamma \varvec{\lambda }\cdot (\varvec{u}_L-\varvec{u}_C) \,\mathrm {d}s -\int _{\Gamma _{N,1} } \bar{\varvec{t}}_l \cdot \varvec{u}_C \,\mathrm {d}s. \end{aligned}$$

Note that, in this case, ${\mathcal {E}}(\varvec{u},d) \approx \widehat{\mathcal {E}}(\varvec{u}_C,\varvec{u}_L,d_L,\varvec{\lambda })$, since the surface integral over the interface $\Gamma$ provides the weak continuity between the local and complementary displacement fields. $\varvec{\lambda }$ is the (unknown) Lagrange multiplier. The corresponding variational problem for $\widehat{\mathcal {E}}$ which *approximates* the reference problem (4) then reads

23 $$\begin{aligned} \underset{ \varvec{u}_C,\varvec{u}_L,d_L }{ \mathrm {argmin} } \,\underset{ \varvec{\lambda } }{\mathrm { max} }\; \widehat{\mathcal {E}}(\varvec{u}_C,\varvec{u}_L,d_L,\varvec{\lambda }). \end{aligned}$$

Owing to condition (21), formulation (23) is called *dual* in the literature, see e.g. [54]. The relation between the solution $(\varvec{u},d)$ of the reference problem (4) and the solution triple $(\varvec{u}_C,\varvec{u}_L,d_L)$ is given by (13) and (14).

*Option 2* One preserves the representations (19) and (20), and, in contrast to (21), imposes only a weak continuity between $\varvec{\lambda }_C$ and $\varvec{\lambda }_L$ on $\Gamma$. The latter is achieved by introducing the functional

24 $$\begin{aligned} {\mathcal {E}}_3(\varvec{u}_\Gamma ,\varvec{\lambda }_C,\varvec{\lambda }_L): = \int _\Gamma \varvec{u}_\Gamma \cdot (\varvec{\lambda }_L+\varvec{\lambda }_C) \,\mathrm {d}s, \end{aligned}$$

with $\varvec{u}_\Gamma \in \mathbf H ^1(\Gamma )$ representing the (unknown) Lagrange multiplier, which has the dimension of a displacement. Summing ${\mathcal {E}}_1$ and ${\mathcal {E}}_2$ with ${\mathcal {E}}_3$, and also regrouping the terms, we finally obtain

25 $$\begin{aligned} \widehat{\mathcal {E}}(\varvec{u}_C,\varvec{u}_L,d_L,\varvec{u}_\Gamma ,\varvec{\lambda }_C,\varvec{\lambda }_L):= & {} \int _{\Omega _C} W(\varvec{\varepsilon }(\varvec{u}_C),0) \,\mathrm {d}{} \mathbf x +\int _{\Omega _L} W(\varvec{\varepsilon }(\varvec{u}_L),d_L) \,\mathrm {d}{} \mathbf x \nonumber \\&+\int _\Gamma \left\{ \varvec{\lambda }_C\cdot (\varvec{u}_\Gamma -\varvec{u}_C) + \varvec{\lambda }_L\cdot (\varvec{u}_\Gamma -\varvec{u}_L) \right\} \mathrm {d}s \nonumber \\&-\int _{\Gamma _{N,1} } \bar{\varvec{t}}_l \cdot \varvec{u}_C \,\mathrm {d}s. \end{aligned}$$

From (25), it can be grasped that the introduction of $\varvec{u}_\Gamma$ enables also to implicitly provide a weak continuity between $\varvec{u}_L$ and $\varvec{u}_C$ across $\Gamma$ via an intermediate function $\varvec{u}_\Gamma$ (this is in addition to the already incorporated weak continuity between $\varvec{\lambda }_L$ and $\varvec{\lambda }_C$). Concluding that ${\mathcal {E}}(\varvec{u},d) \approx \widehat{\mathcal {E}}(\varvec{u}_C,\varvec{u}_L,d_L,\varvec{u}_\Gamma ,\varvec{\lambda }_C,\varvec{\lambda }_L)$, the variational problem for $\widehat{\mathcal {E}}$ in (25) which *approximates* the reference problem (4) will be as follows:

26 $$\begin{aligned} \underset{ \varvec{u}_C,\varvec{u}_L,d_L,\varvec{u}_\Gamma }{ \mathrm {argmin} } \underset{ \varvec{\lambda }_C,\varvec{\lambda }_L }{ \mathrm {max} }\; \widehat{\mathcal {E}}(\varvec{u}_C,\varvec{u}_L,d_L,\varvec{u}_\Gamma ,\varvec{\lambda }_C,\varvec{\lambda }_L). \end{aligned}$$

In this case, the representation of $\varvec{u}$ stemming from the solution of problem (4) in terms of the solution triple $(\varvec{u}_C,\varvec{u}_L,\varvec{u}_\Gamma )$ stemming from (26) reads as

27 $$\begin{aligned} \varvec{u} =\left\{ \begin{array}{ll} \varvec{u}_L, &{} \quad \text { for }{} \mathbf x \in \Omega _L, \\ \varvec{u}_C, &{} \quad \text { for }{} \mathbf x \in \Omega _C, \\ \varvec{u}_\Gamma , &{} \quad \text { for }{} \mathbf x \in \Gamma , \end{array} \right. \end{aligned}$$

whereas the representation for *d* in terms of $d_L$ defined by (14) remains unaltered. In the literature, formulation (26) is sometimes called the *localized Lagrange multipliers* based formulation (we abbreviate this as LLM), where the term ‘localized’ is used to associate the multipliers $\varvec{\lambda }_C,\varvec{\lambda }_L$ and $\varvec{u}_\Gamma$ with the corresponding sub-domains, see e.g. [55–57].

Table 2 briefly summarizes the considered formulations.

**Table 2:** Domain decomposition formulations of the reference problem (4)

**Formulation**	**Imposed continuity between**		**Unknowns**
	$\varvec{u}_{{\varvec{C}}}\,\varvec{ \& }\,\varvec{u}_{{\varvec{L}}}$	$\varvec{\lambda }_{{\varvec{C}}}\,\varvec{ \& }\,\varvec{\lambda }_{{\varvec{L}}}$	
Primal, (18)	Strong	–	$(\varvec{u}_C,\varvec{u}_L,d_L)$
Dual, (23)	Weak	Strong	$(\varvec{u}_C,\varvec{u}_L,d_L,\varvec{\lambda })$
LLM, (26)	Weak	Weak	$(\varvec{u}_C,\varvec{u}_L,d_L,\varvec{u}_\Gamma ,\varvec{\lambda }_C,\varvec{\lambda }_L)$

Formulation (23) is seemingly less computationally demanding than (26), since there is only one extra field $\varvec{\lambda }$ to be solved for in the former case, versus the triple $(\varvec{u}_\Gamma ,\varvec{\lambda }_C,\varvec{\lambda }_L)$ of unknown fields in the latter one. The potential advantage of (26) over (23) is a greater flexibility, at the finite element discretization stage, of handling the interface between complementary and local domains.

As follows, we move on with the LLM formulation (26) and extend it to the *global/local* setting, for which, in turn, a non-intrusive solution procedure is devised. This will lead to a *non-intrusive global/local approach* to the phase-field formulation (4).

### Global/local formulation

As a first step, a so-called fictitious domain $\Omega _F$ is introduced to ‘fill the gap’ obtained in $\Omega$ by removing $\Omega _L$ from it, see Fig. 3b. It is assumed that $\Omega _F$ is constituted by a material with the same linear elastic behaviour as in $\Omega _C$. It is also assumed that $\Omega _F$ is open (i.e. $\Gamma \not \subset \Omega _F$). Unification of $\Omega _F$ with $\Gamma$ and $\Omega _C$ forms the *global* domain $\Omega _G$, that is, $\Omega _G:=\Omega _F\cup \Gamma \cup \Omega _C$. The fictitious domain $\Omega _F$ is furthermore assumed free of geometrical ‘imperfections’ which may be present in $\Omega _L$, see Fig. 3b. Therefore, it is in general $\Omega _G\ne \Omega$, and the constructed global domain $\Omega _G$ should not be confused with the original reference domain $\Omega$.

Summing up the above, the role of the fictitious domain $\Omega _F$ is twofold: it replaces the “sub-regions” of a structure (reference domain) containing geometric details (e.g. holes, inclusions etc.) and/or constitutive non-linearity by there details-free and linearly elastic “counterparts”. The obtained global domain $\Omega _G$ is then straightforwardly suitable for meshing and solving procedures within legacy codes. As it will be also seen below, the use of $\Omega _F$ is essential to realize the concept of *non-intrusiveness* of the computational scheme for solving the coupled global/local formulation.

Next to this, it is assumed that there exists a continuous prolongation of $\varvec{u}_C$ into $\Omega _F$. That is, we introduce a function $\varvec{u}_G\in \mathbf H ^1(\Omega _G)$ such that $\varvec{u}_G|_{\Omega _C}\equiv \varvec{u}_C$ and $\varvec{u}_G=\varvec{u}_C$ on $\Gamma$ in the sense of trace. The former also implies that $\varvec{u}_G=\varvec{0}$ on $\Gamma _{D,0}$ and $\varvec{u}_G=\bar{\varvec{u}}_l$ on $\Gamma _{D,1}$.

Owing to the definitions of $\Omega _G$ and $\varvec{u}_G$, the first term in (25) is recast as follows

$$\begin{aligned} \int _{\Omega _C} W(\varvec{\varepsilon }(\varvec{u}_C),0) \,\mathrm {d}{} \mathbf x= & {} \int _{\Omega _C} W(\varvec{\varepsilon }(\varvec{u}_G),0) \,\mathrm {d}{} \mathbf x \nonumber \\= & {} \int _{\Omega _G} W(\varvec{\varepsilon }(\varvec{u}_G),0) \,\mathrm {d}{} \mathbf x -\int _{\Omega _F} W(\varvec{\varepsilon }(\varvec{u}_G),0) \,\mathrm {d}{} \mathbf x , \end{aligned}$$

and we also substitute $\varvec{u}_G$ for $\varvec{u}_C$ in the third and fourth integrals in (25). This yields the desired *global/local* representation (approximation) of the reference energy functional $\mathcal {E}$ in (1), namely,

28 $$\begin{aligned} \widetilde{\mathcal {E}}(\varvec{u}_G,\varvec{u}_L,d_L,\varvec{u}_\Gamma ,\varvec{\lambda }_C,\varvec{\lambda }_L)&{:=}\int _{\Omega _G} W(\varvec{\varepsilon }(\varvec{u}_G),0) \,\mathrm {d}{} \mathbf x -\int _{\Omega _F} W(\varvec{\varepsilon }(\varvec{u}_G),0) \,\mathrm {d}{} \mathbf x \nonumber \\&+\int _{\Omega _L} W(\varvec{\varepsilon }(\varvec{u}_L),d_L) \,\mathrm {d}{} \mathbf x \nonumber \\&+\int _\Gamma \left\{ \varvec{\lambda }_C\cdot (\varvec{u}_\Gamma -\varvec{u}_G) + \varvec{\lambda }_L\cdot (\varvec{u}_\Gamma -\varvec{u}_L) \right\} \mathrm {d}s \nonumber \\&-\int _{\Gamma _{N,1} } \bar{\varvec{t}}_l \cdot \varvec{u}_G \,\mathrm {d}s. \end{aligned}$$

(We used the $\widetilde{a}$ to distinguish between the previously considered $\widehat{\mathcal {E}}$ and the constructed $\widetilde{\mathcal {E}}$.) The resulting *global/local* variational problem, which approximates the reference formulation (4) reads

29 $$\begin{aligned} \underset{ \varvec{u}_G,\varvec{u}_L,d_L,\varvec{u}_\Gamma }{ \mathrm {argmin} } \underset{ \varvec{\lambda }_C,\varvec{\lambda }_L }{ \mathrm {max} }\; \widetilde{\mathcal {E}}(\varvec{u}_G,\varvec{u}_L,d_L,\varvec{u}_\Gamma ,\varvec{\lambda }_C,\varvec{\lambda }_L), \end{aligned}$$

and the relation between the solution $\varvec{u}$ of (4) and the solution triple $(\varvec{u}_G,\varvec{u}_L,\varvec{u}_\Gamma )$ of (29) is given by

$$\begin{aligned} \varvec{u} =\left\{ \begin{array}{ll} \varvec{u}_L, &{}\quad { for }\, \mathbf x \in \Omega _L, \\ \varvec{u}_G, &{}\quad { for }\, \mathbf x \in \Omega _C, \\ \varvec{u}_\Gamma , &{}\quad { for }\, \mathbf x \in \Gamma . \end{array} \right. \end{aligned}$$

In what follows, for the sake of compactness we set $(\varvec{u}_G,\varvec{u}_L,d_L,\varvec{u}_\Gamma ,\varvec{\lambda }_C,\varvec{\lambda }_L)=:\varvec{z}$.

### Coupled system in weak form

To present the weak formulation of (29), we introduce the directional derivatives of $\widetilde{\mathcal {E}}$ with respect to the various components of $\varvec{z}$. Recalling the function *W* defined by (2), the ‘main’ three derivatives read

30 $$\begin{aligned} \widetilde{\mathcal {E}}_{\varvec{u}_G}(\varvec{z};\varvec{v}_G):= & {} \int _{\Omega _G}\varvec{\sigma }(\varvec{u}_G):\varvec{\varepsilon }(\varvec{v}_G)\,\mathrm {d}{} \mathbf x -\int _{\Omega _F}\varvec{\sigma }(\varvec{u}_G):\varvec{\varepsilon }(\varvec{v}_G)\,\mathrm {d}{} \mathbf x \nonumber \\&\quad -\int _\Gamma \varvec{\lambda }_C \cdot \varvec{v}_G\,\mathrm {d}s -\int _{\Gamma _{N,1} } \bar{\varvec{t}}_l \cdot \varvec{v}_G\,\mathrm {d}s, \end{aligned}$$

where $\varvec{\sigma }(\varvec{u}_G)=\frac{\partial W}{\partial \varvec{\varepsilon }}(\varvec{\varepsilon }(\varvec{u}_G),0)=\frac{\partial \Psi }{\partial \varvec{\varepsilon }}(\varvec{\varepsilon }(\varvec{u}_G))=\mathbb {C}:\varvec{\varepsilon }(\varvec{u}_G)$, and $\varvec{v}_G\in \{ \mathbf H ^1(\Omega _G): \varvec{v}_G=\varvec{0} \; \mathrm {on} \; \Gamma _{D,0}\cup \Gamma _{D,1} \}$ is the test function;

31 $$\begin{aligned} \widetilde{\mathcal {E}}_{\varvec{u}_L}(\varvec{z};\varvec{v}_L):=\int _{\Omega _L} \varvec{\sigma }(\varvec{u}_L,d_L) :\varvec{\varepsilon }(\varvec{v}_L)\,\mathrm {d}{} \mathbf x -\int _\Gamma \varvec{\lambda }_L \cdot \varvec{v}_L\,\mathrm {d}s, \end{aligned}$$

where $\varvec{\sigma }(\varvec{u}_L,d_L)=\frac{\partial W}{\partial \varvec{\varepsilon }}(\varvec{\varepsilon }(\varvec{u}_L),d_L)= (1-d_L)^2\frac{\partial \Psi ^+}{\partial \varvec{\varepsilon }}(\varvec{\varepsilon }(\varvec{u}_L)) +\frac{\partial \Psi ^-}{\partial \varvec{\varepsilon }}(\varvec{\varepsilon }(\varvec{u}_L))$, and $\varvec{v}_L\in \mathbf H ^1(\Omega _L)$ is the test function;

$$\begin{aligned} \widetilde{\mathcal {E}}_{d_L}(\varvec{z};w_L):=\int _{\Omega _L} \left[ -\,2(1-d_L)\Psi ^+(\varvec{\varepsilon }(\varvec{u}_L))w_L +G_c\left( \frac{1}{\ell }d_L w_L + \ell \nabla {d_L}\cdot \nabla {w_L} \right) \right] \,\mathrm {d}{} \mathbf x , \end{aligned}$$

where $w_L\in H^1(\Omega _L)$ is the test function. The following ‘modified’ version of $\widetilde{\mathcal {E}}_{d_L}$, adjusted to account for the irreversibility of the phase-field evolution, will be used in our computations:

32 $$\begin{aligned} \widetilde{\mathcal {E}}^*_{d_L}(\varvec{z};w_L):=\int _{\Omega _L} \left[ -\,2(1-d_L)\mathcal {H}_l(\varvec{\varepsilon }(\varvec{u}_L))w_L +G_c\left( \frac{1}{\ell }d_L w_L + \ell \nabla {d_L}\cdot \nabla {w_L} \right) \right] \,\mathrm {d}{} \mathbf x . \end{aligned}$$

This is similar to the modification discussed for equation (10).

The remaining three variational derivatives of $\widetilde{\mathcal {E}}$ are

33 $$\begin{aligned}&\widetilde{\mathcal {E}}_{\varvec{u}_\Gamma }(\varvec{z};\varvec{v}_\Gamma ):= \int _\Gamma (\varvec{\lambda }_C+\varvec{\lambda }_L) \cdot \varvec{v}_\Gamma \,\mathrm {d}s, \end{aligned}$$

34 $$\begin{aligned}&\widetilde{\mathcal {E}}_{\varvec{\lambda }_C}(\varvec{z};\varvec{\beta }_C):= \int _\Gamma (\varvec{u}_\Gamma -\varvec{u}_G) \cdot \varvec{\beta }_C \,\mathrm {d}s, \end{aligned}$$

35 $$\begin{aligned}&\widetilde{\mathcal {E}}_{\varvec{\lambda }_L}(\varvec{z};\varvec{\beta }_L):= \int _\Gamma (\varvec{u}_\Gamma -\varvec{u}_L) \cdot \varvec{\beta }_L \,\mathrm {d}s, \end{aligned}$$

where $\varvec{v}_\Gamma \in \mathbf H ^1(\Gamma )$ and $\varvec{\beta }_C,\varvec{\beta }_L\in \mathbf L ^2(\Gamma )$ are the corresponding test functions.

Using equations (30) and (31), (32), the global and local weak problems are, respectively, formed:

G $$\begin{aligned}&\int _{\Omega _G}\varvec{\sigma }(\varvec{u}_G):\varvec{\varepsilon }(\varvec{v}_G)\,\mathrm {d}{} \mathbf x -\int _{\Omega _F}\varvec{\sigma }(\varvec{u}_G):\varvec{\varepsilon }(\varvec{v}_G)\,\mathrm {d}{} \mathbf x -\int _\Gamma \varvec{\lambda }_C \cdot \varvec{v}_G\,\mathrm {d}s \nonumber \\&\quad -\int _{\Gamma _{N,1} } \bar{\varvec{t}}_l \cdot \varvec{v}_G\,\mathrm {d}s =0, \end{aligned}$$

and

L $$\begin{aligned} \left\{ \begin{array}{l} \displaystyle \int _{\Omega _L}\varvec{\sigma }(\varvec{u}_L,d_L):\varvec{\varepsilon }(\varvec{v}_L)\,\mathrm {d}{} \mathbf x -\int _\Gamma \varvec{\lambda }_L \cdot \varvec{v}_L\,\mathrm {d}s=0, \\ \displaystyle \int _{\Omega _L}\left[ -2(1-d_L)\mathcal {H}_l(\varvec{\varepsilon }(\varvec{u}_L))w_L+G_c\left( \frac{1}{\ell }d_L w_L + \ell \nabla {d_L}\cdot \nabla {w_L} \right) \right] \,\mathrm {d}{} \mathbf x =0, \end{array} \right. \end{aligned}$$

whereas equations (33), (34) and (35) are used for establishing the (weak) coupling between them: 
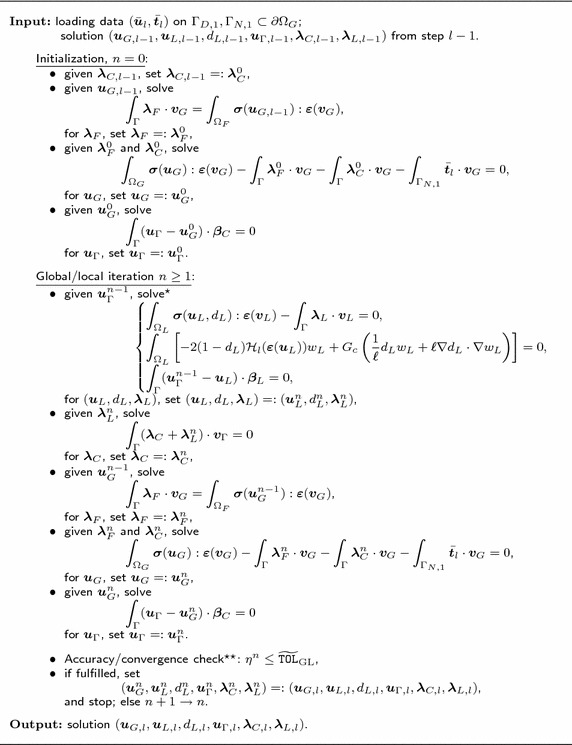
 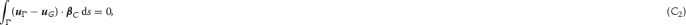 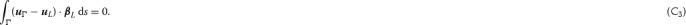 Here, $(\varvec{u}_G,\varvec{u}_L,d_L,\varvec{u}_\Gamma ,\varvec{\lambda }_C,\varvec{\lambda }_L)$ is the vector of the unknowns to be solved for, and $(\varvec{v}_G,\varvec{v}_L,w_L,$ $\varvec{v}_\Gamma ,\varvec{\beta }_C,\varvec{\beta }_L)$ is the vector of the corresponding test functions.

For the presented system of equations, a computational scheme can already be devised. We should notice, however, that equation (G) in the current form does not fit in the notion of non-intrusiveness yet. Indeed, being a linear one, it can naturally be solved for $\varvec{u}_G$ ‘straightforwardly’. But the presence of the two domain integrals, namely, over $\Omega _G$ and $\Omega _F\subset \Omega _G$ would imply in this case the need to simultaneously access the corresponding stiffness matrices (in the following, $\mathsf {K}_G$ and $\mathsf {K}_F$), or, in other words, a necessity of modifying $\mathsf {K}_G$—a situation that contradicts the concept of non-intrusiveness. Avoiding this can be done in two steps: first, by introducing a partitioning of equation (G), and then, devising the appropriate iterative solution procedure. The former will be presented here, and the latter is addressed in “Non-intrusive computational scheme” section.

**Fig. 4:**
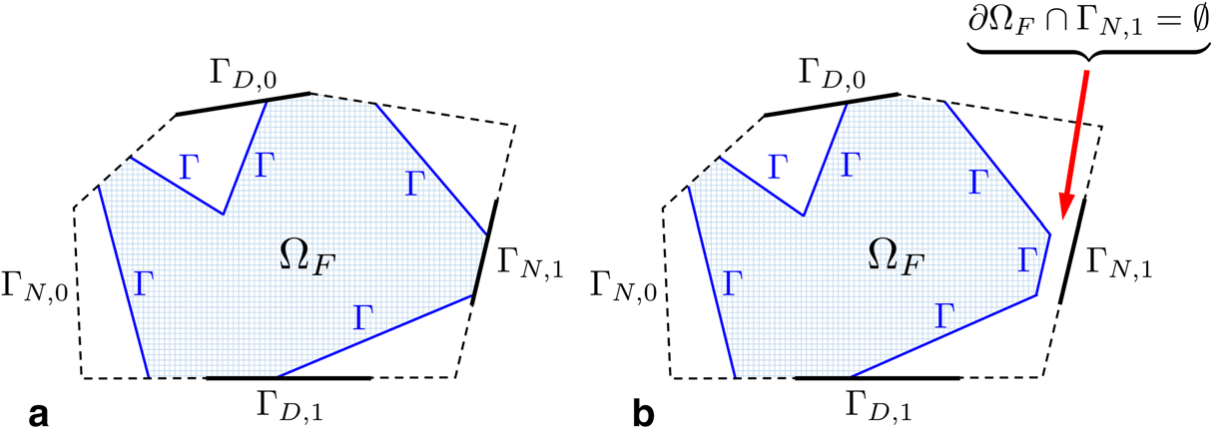
**a** The possible complex nature of $\partial \Omega _F$ illustrating equation (37); **b** choice of $\Omega _L$ that results in $\partial \Omega _F\cap \Gamma _{N,1}=\emptyset$ for $\Omega _F$

We focus on the domain integral over $\Omega _F$ in (G). The divergence theorem leads to

36 $$\begin{aligned} \int _{\Omega _F}\varvec{\sigma }(\varvec{u}_G):\varvec{\varepsilon }(\varvec{v}_G)\,\mathrm {d}{} \mathbf x =-\int _{\Omega _F}\mathrm {div}(\varvec{\sigma }(\varvec{u}_G))\cdot \varvec{v}_G\,\mathrm {d}{} \mathbf x +\int _{\partial \Omega _F} \varvec{\sigma }(\varvec{u}_G)\cdot \varvec{n}_{\partial \Omega _F} \cdot \varvec{v}_G\,\mathrm {d}s, \end{aligned}$$

where $\varvec{n}_{\partial \Omega _F}$ is the unit outward normal vector to $\partial \Omega _F$. The first term in the right-hand side of (36) can be canceled using the divergence-free assumption for the stress (no body forces in $\Omega _F$). The second term can be simplified as follows. In the most general case, $\partial \Omega _F$ is composed of five non-overlapping parts, including $\Gamma$. More precisely,

37 $$\begin{aligned} \partial \Omega _F=\Gamma \cup (\partial \Omega _F\cap \Gamma _{N,0}) \cup (\partial \Omega _F\cap \Gamma _{D,0}) \cup (\partial \Omega _F\cap \Gamma _{N,1}) \cup (\partial \Omega _F\cap \Gamma _{D,1}), \end{aligned}$$

as sketched in Fig. 4a, and hence

38 $$\begin{aligned} \int _{\partial \Omega _F} R =\int _\Gamma R +\int _{\partial \Omega _F\cap \Gamma _{N,0}} R +\int _{\partial \Omega _F\cap \Gamma _{D,0}} R +\int _{\partial \Omega _F\cap \Gamma _{N,1}} R +\int _{\partial \Omega _F\cap \Gamma _{D,1}} R,\!\!\!\! \end{aligned}$$

with $R:=\varvec{\sigma }(\varvec{u}_G)\cdot \varvec{n}_{\partial \Omega _F} \cdot \varvec{v}_G$. In this case, due to the following basic properties

- $$\varvec{\sigma }(\varvec{u}_G)\cdot \varvec{n}_{\Gamma _{N,0}}=0$$ on $$\Gamma _{N,0}$$,
- $\varvec{v}_G=0$ on $\Gamma _{D,0}$ and on $\Gamma _{D,1}$,
- $$\varvec{\sigma }(\varvec{u}_G)\cdot \varvec{n}_{\Gamma _{N,1}}=\bar{\varvec{t}}_l$$ on $$\Gamma _{N,1}$$,

the corresponding integrals in the right-hand side of (38) are simplified, thus yielding

$$\begin{aligned} \int _{\partial \Omega _F} \varvec{\sigma }(\varvec{u}_G)\cdot \varvec{n}_{\partial \Omega _F} \cdot \varvec{v}_G\,\mathrm {d}s =\int _\Gamma \varvec{\sigma }(\varvec{u}_G)\cdot \varvec{n}_\Gamma \cdot \varvec{v}_G\,\mathrm {d}s +\int _{\partial \Omega _F\cap \Gamma _{N,1}} \bar{\varvec{t}}_l \cdot \varvec{v}_G\,\mathrm {d}s. \end{aligned}$$

Here, $\varvec{n}_\Gamma$ is the unit normal vector on $\Gamma$, outward with respect to $\Omega _F$. To further simplify the last expression, we note that it is always possible to pick $\Omega _L$ (and, hence, the resulting $\Omega _F$) such that $\partial \Omega _F\cap \Gamma _{N,1}=\emptyset$, see Fig. 4b, and, as a result, the last surface integral cancels. For (36), this eventually yields:

39 $$\begin{aligned} \int _{\Omega _F}\varvec{\sigma }(\varvec{u}_G):\varvec{\varepsilon }(\varvec{v}_G)\,\mathrm {d}{} \mathbf x =\int _\Gamma \varvec{\sigma }(\varvec{u}_G)\cdot \varvec{n}_\Gamma \cdot \varvec{v}_G\,\mathrm {d}s. \end{aligned}$$

It can now be assumed that, given $\varvec{u}_G$, there exists $\varvec{\lambda }_F\in \mathbf L ^2(\Gamma )$ such that

40 $$\begin{aligned} \int _\Gamma \varvec{\lambda }_F \cdot \varvec{v}_G\,\mathrm {d}s =\int _\Gamma \varvec{\sigma }(\varvec{u}_G)\cdot \varvec{n}_\Gamma \cdot \varvec{v}_G\,\mathrm {d}s, \end{aligned}$$

holds. In the above, we use the subscript *F* to indicate, according to (39), the relation of the corresponding quantity to $\Omega _F$ (more precisely, to the restriction of $\varvec{u}_G$ to $\Omega _F$).

Owing to (39) and (40), we finally arrive at the following *partitioned* representation of equation (G): 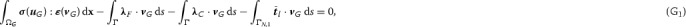 with $$\varvec{\lambda }_F$$ satisfying 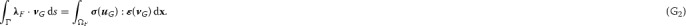


Equations ($\hbox {G}_1$), ($\hbox {G}_2$), system (L) and coupling equations ($\hbox {C}_1$), ($\hbox {C}_2$), ($\hbox {C}_3$) constitute what we term *global/local coupled system*, which is to be solved for the vector $(\varvec{u}_G,\varvec{u}_L,d_L,\varvec{u}_\Gamma ,\varvec{\lambda }_C,\varvec{\lambda }_L)$.

### Non-intrusive computational scheme

Let $n\ge 0$ be the iteration index. For designing at a fixed loading step *l* the iterative solution procedure for the global/local system defined by ($\hbox {G}_1$), ($\hbox {G}_2$), (L), and ($\hbox {C}_1$), ($\hbox {C}_2$), ($\hbox {C}_3$), the following prerequisites are taken into account:

- Since the data $(\bar{\varvec{u}}_{l},\bar{\varvec{t}}_{l})$ are posed on $\Gamma _{D,1},\Gamma _{N,1}\subset \partial \Omega _G$, the process initialization (i.e. iteration $n=0$) is started with the solution of global problem ($\hbox {G}_1$), ($\hbox {G}_2$).
- In order to fit equation ($\hbox {G}_1$) with $\varvec{\lambda }_F=\varvec{\lambda }_F(\varvec{u}_G)$ in the concept of non-intrusiveness, $\varvec{\lambda }_F$ must be treated as a known quantity. This defines the order in which equations ($\hbox {G}_1$) and ($\hbox {G}_2$) are solved at any iteration $n\ge 0$: the solution of ($\hbox {G}_2$) precedes the solution of ($\hbox {G}_1$). In this case, as desired, the stiffness matrix $\mathsf {K}_G$ remains unaltered; the access to $\mathsf {K}_F$ is still required, but only at the stage of solving ($\hbox {G}_2$), not ($\hbox {G}_1$).
- For solving ($\hbox {G}_1$), $\varvec{\lambda }_C$ must be also known. At $n=0$, $\varvec{\lambda }_C$ can simply be taken from the previous loading step. At $n\ge 1$, we use coupling equation ($\hbox {C}_1$) for the extraction of $\varvec{\lambda }_C$, assuming $\varvec{\lambda }_L$ is already known. This defines the order in which the global and local problems are solved: at any iteration starting from $n=1$, the solution of (L) precedes the solution of ($\hbox {G}_1$).
  We also notice that:
- Coupling equation ($\hbox {C}_3$) provides the boundary condition for $\varvec{u}_L$ of the local problem (L).
- Coupling equation ($\hbox {C}_2$) is used for the recovery of $\varvec{u}_\Gamma$.

As follows from (c) and (e), elimination of $\varvec{\lambda }_C$ and $\varvec{u}_\Gamma$ from the set of unknowns to be originally solved for is achieved. These two quantities, as well as $\varvec{\lambda }_F$, are the recovered ones.

The summary of the solution operations to be performed at any iteration *n* of the procedure, excluding the initialization step ($n=0$), is as follows:

- solution of local problem (L) coupled with ($\hbox {C}_3$),
- recovery phase using ($\hbox {C}_1$) and ($\hbox {G}_2$),
- solution of global problem ($\hbox {G}_1$),
- recovery phase using ($\hbox {C}_2$).

The detailed scheme, including the iteration $n=0$, is depicted in Table 3. Note that in all equations in the table we omit $\mathrm {d}{} \mathbf x$ and $\mathrm {d}s$.

**Table 3:** Non-intrusive iterative solution process for ($\hbox {G}_1$), ($\hbox {G}_2$), (L), and ($\hbox {C}_1$), ($\hbox {C}_2$), ($\hbox {C}_3$) at a fixed loading step *l*

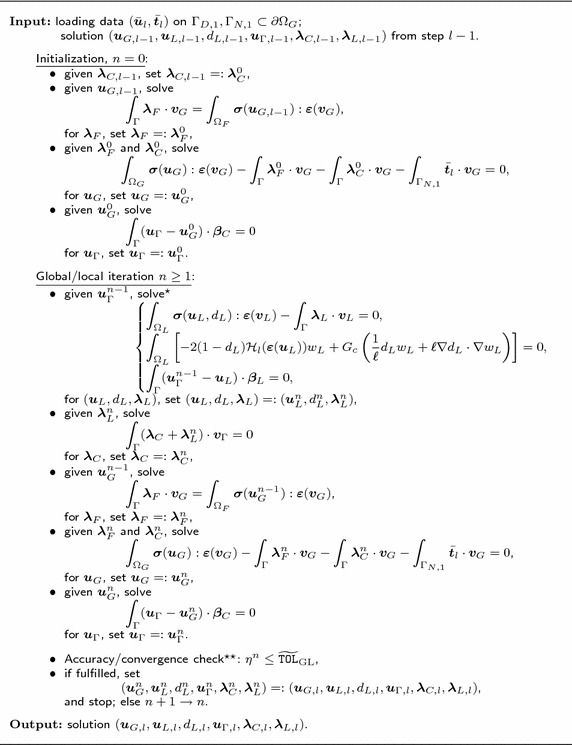	

* See “Staggered process for the local problem” section, ** see “Accuracy/convergence check” section

#### Staggered process for the local problem

Solution of the local system in Table 3 at the given global/local iteration $n\ge 1$ requires an additional nested iterative solution process. In our case, this is the staggered procedure from Table 1, which is adjusted to handle an extra variable $\varvec{\lambda }_L$, and is also equipped with the appropriate definition of the input (initial guess) data and of the stopping criterion.

The initial guess for the staggered loop (with the iteration index $k\ge 0$) is chosen as follows. At iteration $n=1$ (and staggered iteration $k=0$) the values $(\varvec{u}_{L,l-1},d_{L,l-1},\varvec{\lambda }_{L,l-1})$ known from the previous loading step are used as the initial guess. At $n\ge 2$ (and staggered iteration $k=0$), we naturally take $(\varvec{u}_L^{n-1},d_L^{n-1},\varvec{\lambda }_L^{n-1})$.

At any fixed iteration $n\ge 1$, the accuracy check for the solution triple $(\varvec{u}_L^{(k)},d_L^{(k)},\varvec{\lambda }_L^{(k)})$ obtained at the staggered iteration $k\ge 0$ is performed as follows:

41 $$\begin{aligned} \mathrm {Res}_\mathrm {Stag}^k:=|\widetilde{\mathcal {E}}_{\varvec{u}_L}(\varvec{u}_L^{(k)},d_L^{(k)},\varvec{\lambda }_L^{(k)};\varvec{v}_L)|\le \texttt {TOL}_\mathrm {Stag}, \quad \forall \; \varvec{v}_L\in \mathbf H ^1(\Omega _L), \end{aligned}$$

where, to recall, $\widetilde{\mathcal {E}}_{\varvec{u}_L}$ is given by (31). If (41) is fulfilled—note again that in the following numerical test, $\texttt {TOL}_\mathrm {Stag}:=10^{-5}$ – the staggered process is stopped, we set $(\varvec{u}_L^{(k)},d_L^{(k)},\varvec{\lambda }_L^{(k)})=:(\varvec{u}_L^n,d_L^n,\varvec{\lambda }_L^n)$ and perform $n+1\rightarrow n$.

#### Accuracy/convergence check

Derivation of the convergence and stopping criteria for the global/local iterative solution process in Table 3 is rather straightforward. Indeed, at any iteration $n\ge 1$, the solution outcome is denoted as $(\varvec{u}_G^n,\varvec{u}_L^n,d_L^n,\varvec{u}_\Gamma ^n,\varvec{\lambda }_C^n,\varvec{\lambda }_L^n)$. Plugging this in equations ($\hbox {G}_1$), ($\hbox {G}_2$), (L), ($\hbox {C}_1$), ($\hbox {C}_2$), ($\hbox {C}_3$) and comparing the obtained outcome with the corresponding equations in Table 3, it is straightforward to locate the imbalanced quantities:

42 $$\begin{aligned} \int _\Gamma (\varvec{u}_\Gamma ^n-\varvec{u}_L^n) \cdot \varvec{\beta }_L \ne 0, \end{aligned}$$

and

43 $$\begin{aligned} \int _\Gamma \varvec{\lambda }_F^n \cdot \varvec{v}_G \ne \int _{\Omega _F}\varvec{\sigma }(\varvec{u}_G^n):\varvec{\varepsilon }(\varvec{v}_G). \end{aligned}$$

Therefore, the solution accuracy at *n* is measured by the quantity

44 $$\begin{aligned} \mathrm {Res}_\mathrm {GL}^n:= \mathrm {Res}_\mathrm {Stag}^{\prime \mathrm {last}\; k^{\prime }} +|\int _\Gamma (\varvec{u}_\Gamma ^n-\varvec{u}_L^n) \cdot \varvec{\beta }_L| +|\int _\Gamma (\varvec{\lambda }_F^n-\varvec{\lambda }_F^{n+1}) \cdot \varvec{v}_G|, \quad \forall \; \varvec{\beta }_L,\varvec{v}_G, \end{aligned}$$

where $\mathrm {Res}_\mathrm {Stag}$ is the staggered residual of the local problem with ‘last *k*’ denoting the index of the converged staggered solution (see “Staggered process for the local problem” section for details), and $\lambda _F^{n+1}$ is recovered (post-processed) from the right-hand side of (43). The stopping criterion for the global/local loop can then be defined as

45 $$\begin{aligned} \mathrm {Res}_\mathrm {GL}^n\le \texttt {TOL}_\mathrm {GL}, \end{aligned}$$

with $\texttt {TOL}_\mathrm {GL}$ to be prescribed. Owing to the ‘nested in’ nature of the staggered process, it has to be $\texttt {TOL}_\mathrm {Stag}<\texttt {TOL}_\mathrm {GL}$. Recalling that $\texttt {TOL}_\mathrm {Stag}=10^{-5}$, we set $\texttt {TOL}_\mathrm {GL}:=10^{-4}$.

In our computations (see Table 3), we use a more convenient form of the stopping criterion. Setting

$$\begin{aligned} \eta _{\varvec{u}}^n:=\left\| \varvec{u}_\Gamma ^n-\varvec{u}_L^n \right\| _\mathbf{L ^2(\Gamma )}, \quad \eta _{\varvec{\lambda }}^n:=\left\| \varvec{\lambda }_F^n-\varvec{\lambda }_F^{n+1} \right\| _\mathbf{L ^2(\Gamma )}, \end{aligned}$$

we define $\eta ^n:=\sqrt{ (\eta _{\varvec{u}}^n)^2 + (\eta _{\varvec{\lambda }}^n)^2 }$, and use this quantity to now check

$$\begin{aligned} \eta ^n \le \widetilde{\texttt {TOL}}_\mathrm {GL}:=10^{-6}. \end{aligned}$$

This choice of $\widetilde{\texttt {TOL}}_\mathrm {GL}$ fulfills the requirement $\eta ^n\overset{!}{<}{} \texttt {TOL}_\mathrm {GL}-\texttt {TOL}_\mathrm {Stag}$, which is stipulated by (44), (45), and the already prescribed above magnitudes of $\texttt {TOL}_\mathrm {GL}$ and $\texttt {TOL}_\mathrm {Stag}$.

Since the quantity $\eta$ naturally stems from the global/local solution accuracy check $\widetilde{\mathcal {E}}_{\varvec{z}}(\varvec{z}^n;\varvec{y})=0$, it represents not only the *iterative convergence* indicator, but also the *solution accuracy* indicator—a very desired property, since the former is only suitable for tracing the convergence of the corresponding iterative solution process, but, clearly, is not adequate for stopping criterion. The corresponding ingredients $\eta _{\varvec{u}}$ and $\eta _{\varvec{\lambda }}$ are only iterative convergence indicators, but none of them provides an adequate check of the solution accuracy. In particular, since $\eta _{\varvec{u}}$ measures, though implicitly, the *displacement continuity*—a match between $\varvec{u}_G$ and $\varvec{u}_L$across $\Gamma$ (recall that the *traction continuity*—a match between $\varvec{\lambda }_C$ and $\varvec{\lambda }_L$ on $\Gamma$—is, in our case, fulfilled automatically), it is also the indicator of a good “gluing” between the two models.

#### Incremental setting

For later developments (“Results and discussion” section), it proves convenient to reformulate the global equation in incremental form. It is straightforward to see that for a given global/local iteration $n\ge 1$, this reads: given the triple $(\varvec{u}_G^{n-1},\varvec{\lambda }_F^{n-1},\varvec{\lambda }_C^{n-1})$ known from the iteration $n-1$, as well as $(\varvec{\lambda }_F^n,\varvec{\lambda }_C^n)$ ‘recovered’ at the iteration *n*, we solve 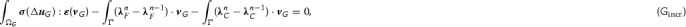


for $\Delta \varvec{u}_G=:(\Delta \varvec{u}_G)^n$ and set

46 $$\begin{aligned} \varvec{u}_G^n:=\varvec{u}_G^{n-1}+(\Delta \varvec{u}_G)^n. \end{aligned}$$

We term equation (46) a ‘direct update’ within the global/local iterative procedure. This is in contrast to the notion of a ‘relaxed/accelerated update’ to be considered in Section .

### Finite element discretization

In the following, for the sake of simplicity, we assume the dimension of the reference problem is 2. Let $\mathcal {P}$ be a finite element partition of $\Omega$ into triangles or quadrilaterals, *I* be the number of nodes in $\mathcal {P}$, and $N_i$, $i=1,\ldots ,I$ be the nodal shape function associated with the node *i* and supported on the collection of elements in $\mathcal {P}$ that share *i*. Finally, let a scalar-valued quantity $\hat{\cdot }_i$ represent the nodal value.

The standard discretization of the solution of the reference problem (using Voigt’s notation) is as follows:

$$\begin{aligned} \varvec{u} = \left[ \begin{array}{c} u_x\\ u_y \end{array}\right] = \sum _{i=1}^I \left[ \begin{array}{cc} N_i&{}0\\ 0&{}N_i \end{array} \right] \left[ \begin{array}{c} \hat{u}_{x,i}\\ \hat{u}_{y,i} \end{array}\right] =:\varvec{N}_u \hat{\varvec{u}}, \end{aligned}$$

where $\varvec{N}_u$ is the $2\times 2I$ interpolation/basis matrix and $\varvec{u}$ is the $2I\times 1$ displacement nodal-vector, and

$$\begin{aligned} d = \sum _{i=1}^I N_i \hat{d}_i=:\varvec{N}_d \hat{\varvec{d}}. \end{aligned}$$

where $\varvec{N}_d$ is the $1\times I$ interpolation/basis matrix and *d* is the $I\times 1$ phase-field nodal-vector. $N_i$ constitutes both matrices $\varvec{N}_u$ and $\varvec{N}_d$. The corresponding representation of the gradients reads:

$$\begin{aligned} \varvec{\varepsilon }(\varvec{u}) = \left[ \begin{array}{c} \varepsilon _{xx}\\ \varepsilon _{yy} \\ \varepsilon _{xy} \end{array}\right] = \sum _{i=1}^I \left[ \begin{array}{cc} \frac{\partial N_i}{\partial x}&{}0 \\ 0&{}\frac{\partial N_i}{\partial y} \\ \frac{1}{2}\frac{\partial N_i}{\partial y}&{} \frac{1}{2}\frac{\partial N_i}{\partial x} \end{array} \right] \left[ \begin{array}{c} \hat{u}_{x,i}\\ \hat{u}_{y,i} \end{array}\right] =:\varvec{B}_u\hat{\varvec{u}}, \end{aligned}$$

where $\varvec{B}_u$ is a $3\times 2I$ matrix, and

$$\begin{aligned} \nabla d = \left[ \begin{array}{c} \frac{\partial d}{\partial x}\\ \frac{\partial d}{\partial y} \end{array}\right] = \sum _{i=1}^I \left[ \begin{array}{cc} \frac{\partial N_i}{\partial x} \\ \frac{\partial N_i}{\partial y} \end{array} \right] \hat{d}_i =:\varvec{B}_d \hat{\varvec{d}}, \end{aligned}$$

where $\varvec{B}_d$ is a $2\times I$ matrix. The problem test functions $\varvec{v}$ and *w*, as well as their gradients are discretized accordingly.

For the global/local formulation, we assume the existence of the partitions $\mathcal {P}_G$ and $\mathcal {P}_L$ of $\Omega _G$ and $\Omega _L$, respectively. Using this and adopting the above notations, the solution discretizations are given by

47 $$\begin{aligned} \varvec{u}_G=\varvec{N}_u^G\hat{\varvec{u}}_G, \quad \varvec{u}_L=\varvec{N}_u^L\hat{\varvec{u}}_L, \quad d_L=\varvec{N}_d^L\hat{\varvec{d}}_L, \end{aligned}$$

such that

48 $$\begin{aligned} \varvec{\varepsilon }(\varvec{u}_G)=\varvec{B}_u^G\hat{\varvec{u}}_G, \quad \varvec{\varepsilon }(\varvec{u}_L)=\varvec{B}_u^L\hat{\varvec{u}}_L, \quad \nabla d_L=\varvec{B}_d^L\hat{\varvec{d}}_L. \end{aligned}$$

Note that the superscripts *G* and *L* are introduced in the corresponding definitions of $\varvec{N}_u$, $\varvec{N}_d$ and $\varvec{B}_u$, $\varvec{B}_d$.

To construct the discretization of the Lagrange multipliers $\varvec{\lambda }_C$, $\varvec{\lambda }_L$, $\varvec{u}_\Gamma$ and the supplementary quantity $\varvec{\lambda }_F$ on $\Gamma$ we account for the following. In the most general situation, three distinct partitions of $\Gamma$ may be assumed: the restrictions of $\mathcal {P}_G$ and $\mathcal {P}_L$—denoted as $\mathcal {T}_G$ and $\mathcal {T}_L$, respectively—which serve to create the corresponding bases for $\varvec{\lambda }_C$ (also $\varvec{\lambda }_F$) and $\varvec{\lambda }_L$, and the ‘independent’ partition $\mathcal {T}_\Gamma$ to be used for creating the basis for $\varvec{u}_\Gamma$. Introducing the three related basis matrices $\varvec{N}_\lambda ^G$, $\varvec{N}_\lambda ^L$ and $\varvec{N}_u^\Gamma$, we write

49 $$\begin{aligned} \varvec{\lambda }_C=\varvec{N}_\lambda ^G\hat{\varvec{\lambda }}_C, \quad \varvec{\lambda }_L=\varvec{N}_\lambda ^L\hat{\varvec{\lambda }}_L, \quad \varvec{u}_\Gamma =\varvec{N}_u^\Gamma \hat{\varvec{u}}_\Gamma , \quad \varvec{\lambda }_F=\varvec{N}_\lambda ^G\hat{\varvec{\lambda }}_F. \end{aligned}$$

In the following, for our numerical example, we assume that:

- the partitions $\mathcal {T}_G$, $\mathcal {T}_L$ and $\mathcal {T}_\Gamma$ match (this is usually termed a ‘matching case’);
- the basis in the global and local domains is identical, that is, $\varvec{N}_u^G=\varvec{N}_u^L=:\varvec{N}_u$;
- the basis on the interface is obtained from $\varvec{N}_u$ by the corresponding restriction, that is, $\varvec{N}_\lambda ^G=\varvec{N}_\lambda ^L=\varvec{N}_u^\Gamma =\varvec{N}_u|_\Gamma$;
- the nodal shape functions $N_i$ composing bases $\varvec{N}_u$ and $\varvec{N}_d$ are piecewise linear.

Because of the matching interface situation, no intricate data transfer methods (the construction of prolongation and restriction operators, generalized inverse matrices etc.) are required. Also, with the above choice of the discretization basis for the Lagrange multipliers, the related *inf-sup* condition is fulfilled, see e.g. [58, 59].

**Fig. 5:**
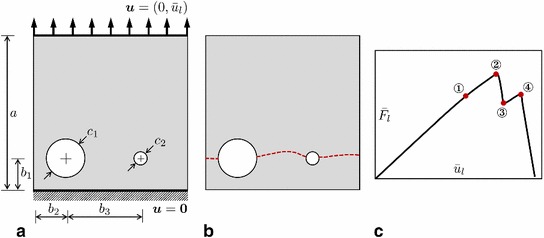
**a** Specimen geometry and loading conditions; sketches of (**b**) the fracture pattern and **c** the load-displacement curve with the points of interest

Using expressions (47), (48) and (49) along with the above assumptions, the matrix representation of all equations in Table 3 is straightforward.

## Results and discussion

To illustrate the proposed approach, we consider the following benchmark problem. A square specimen with two holes of different diameters is subjected to tension loading, see Fig. 5a. The holes are introduced to weaken the structure and to facilitate the specimen cracking in absence of a stronger singularity such as a pre-existing crack. The holes location is chosen such that prediction of the sub-region where cracking occurs (hence, the local domain for the forthcoming global/local analysis) is feasible. Taking a different size of the holes is intended to obtain a geometrically non-trivial crack pattern, as depicted in Fig. 5b. This, moreover, results in a multi-stage crack propagation process to be manifested by a load-displacement response with two peak points, see Fig. 5c for a sketch, and Figs. 7 and 12 for the actually obtained results. We believe that the present setup, being neither extremely complex, nor trivial, is suitable for the purpose of a qualitative and quantitative comparison between the reference results and results obtained with the proposed global/local approach.

The geometric data are as follows (all given in mm): $a=1$, $b_1=0.197$, $b_2=0.210$, $b_3=0.490$ with the hole diameters $c_1=0.247$ and $c_2=0.0806$. The material data are: Young’s modulus $E=210\,\mathrm {GPa}$, Poisson’s ratio $\nu =0.3$ and the critical energy release rate $G_c=2.7\cdot 10^{-3}\,\mathrm {kN/mm}$. The characteristic length in the phase-field formulation is $\ell =1.5\cdot 10^{-2}\,\mathrm {mm}$. We consider the plane-strain situation.

The algorithmic parameters are: the loading $\bar{u}_l=l\Delta u$ with $l\in [1,110)$ and the increment size $\Delta u:= 0.06\cdot 10^{-3}\,\mathrm {mm}$, the tolerance magnitudes are $\mathtt {TOL}_\mathrm {NR}:=10^{-8}$, $\mathtt {TOL}_\mathrm {Stag}:=10^{-5}$, and $\widetilde{\texttt {TOL}}_\mathrm {GL}:=10^{-6}$.

**Fig. 6:**
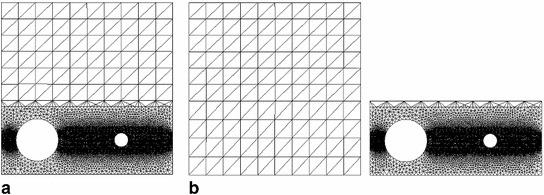
Finite element mesh used for the discretization **a** of the reference domain $\Omega$, **b** of the global and local domains $\Omega _G$ and $\Omega _L$, respectively

We recall that we use $P_1$-triangles for approximating all unknown variables both in the reference and global/local formulations. The minimum finite element size in the reference and local domains is 0.004 mm, the maximum element size in the reference and global domains is $0.1\sqrt{2}$ mm. The former fulfills the heuristic requirement $h<\ell /2$ for the element size inside the localization zone (i.e. the support) of *d*. The reference domain partition contains 18,672 elements. The discretizations of the global and local domains contain 200 and 18,552 elements, respectively. That is, in our case, the reference and global/local problems have a comparable discretization size, as can be grasped from Fig. 6.

### Reference and global/local results

**Fig. 7:**
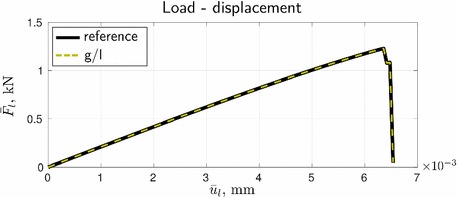
Comparison of the load-displacement curves

**Fig. 8:**
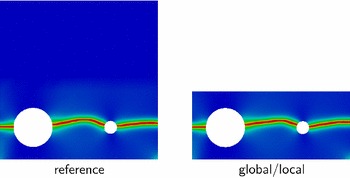
Comparison of the computed phase-field profiles

We start here with the presentation of the quantitative and qualitative reference and global/local results and their comparison. As desired, the two load-displacement curves in Fig. 7 are identical in the entire range of loading, including the pre- and post-peak behavior. The computed phase-field profiles in Fig. 8 are also in a very good agreement. This is already a good indicator of the potential of the global/local approach with application to systems with strong non-linearity and localization.

**Fig. 9:**
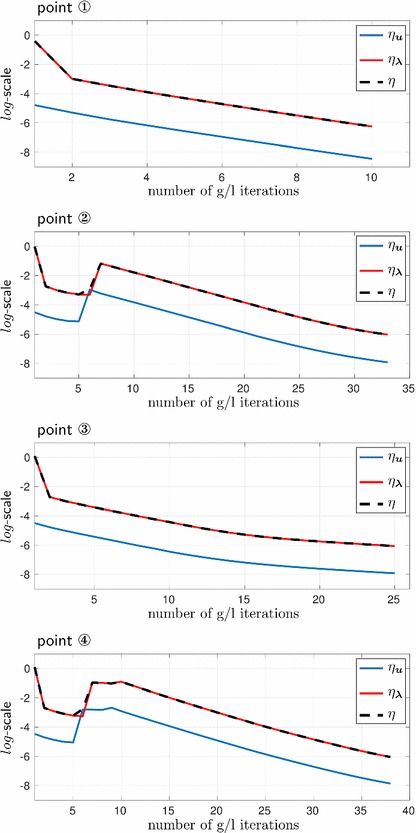
Convergence behavior of the global/local iterative solution process at four different loading steps (the points 1–4 from Fig. 5c), illustrated in terms of the indicator $\eta$, as well as its ingredients $\eta _{\varvec{u}}$ and $\eta _{\varvec{\lambda }}$

For a better insight into the the iterative convergence behavior of the global/local solution process, in Fig. 9 we depict the convergence indicators from “Accuracy/convergence check” section for four given loading steps corresponding to the points 1–4 of our interest sketched in Fig. 5c. Thus, we plot the quantities $\eta _{\varvec{u}}$, $\eta _{\varvec{\lambda }}$ and $\eta =\sqrt{\eta _{\varvec{u}}^2+\eta _{\varvec{\lambda }}^2}$ such that the amount of global/local iterations required for the solution convergence at the step (also in comparison with other steps) can be detected.

The first important observation is that $\eta _{\varvec{u}}$, which implicitly measures the displacement discontinuity between the solutions of the global and local problem across the interface, is two orders of magnitude less than $\eta _{\varvec{\lambda }}$. Thus, its contribution to $\eta$, which is used not only for tracing the convergence of the iterative solution process, but also for the solution accuracy check, is negligible. This means that a stopping criterion based solely on the use of $\eta _{\varvec{u}}$ (what seems typical for the global/local approaches in e.g. plasticity) will yield, in our case, erroneous results. Secondly, it can be noted that a quite large amount of global/local iterations is needed, especially at loading steps corresponding to the peak loads of the load-displacement curves in Fig. 7 (the points of interest 2 and 4 from Fig. 5c).

**Fig. 10:**
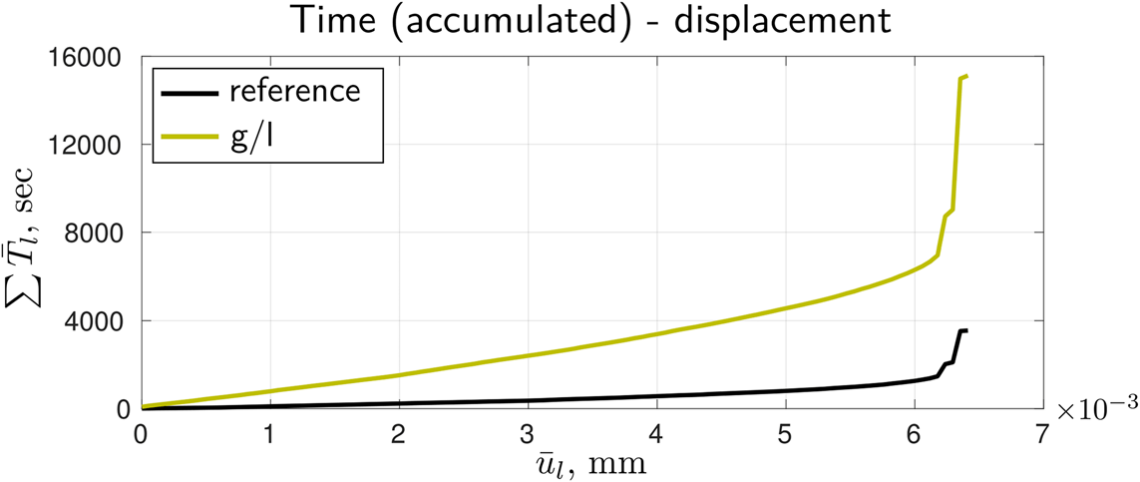
Time-displacement curves in terms of ‘accumulated time’

**Fig. 11:**
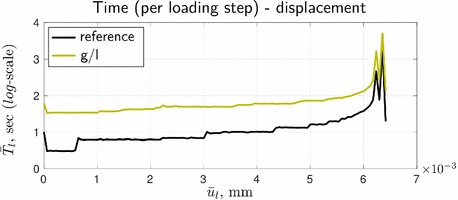
Time-displacement curves in terms of ‘time per loading step’

Resulting from the slow convergence of the global/local procedure, the corresponding cumulative computational time turns out to be high, see Fig. 10, where also the time for solving the reference formulation by the staggered scheme is depicted. For the given setup, with a standard machine (Intel(R) Core(TM) i7-3770 OK, CPU 3.5 GHz, RAM 16.0 GB) it takes about one hour of staggered computations vs. approximately four hours required for the global/local approach. (We should note however that our goal was not to gain computational efficiency, but rather to enable computations with legacy codes.) High efforts are not surprising, as the global/local problem has a larger discretization size than the reference problem, and three nested iterative processes vs. two for the reference problem. The latter results in a larger time per loading step, as can be seen in Fig. 11.

It can be grasped that the rapid increase of cumulative time in Fig. 10 for both formulation appears at loading steps related to the peak points 2 and 4. Also, regardless of the formulation, the computational time per step in Fig. 11 at these points is significantly higher (by almost two orders of magnitude, to be more precise) than at the pre-peak loading steps. These observations correlate with the convergence results from Fig. 9.

Non-convexity and non-linearity of the global/local formulation, as well as the complicated multi-level iterative nature of the related iterative solution procedure result in a generically slow convergence of the approach. Another impacting factor that should be noted is that the stiffness matrix of the global problem $\mathsf {K}_G$ is never updated within the global/local computation process. Incorporation of an incremental update relaxation in this process is thus our next goal, with the objective to obtain an acceleration of the convergence process.

### Relaxation/acceleration techniques: Aitken’s, SR1, Broyden et al.

Following [39] and [51], we will consider and incorporate two types of relaxation/acceleration techniques into our approach: Aitken’s $\Delta ^2$-method (also known as dynamic relaxation, whose efficient implementation in fluid-structure interaction computations has already been reported [60, 61]) and Quasi-Newton correction. Within the family of Quasi-Newton correction formulae, we restrict ourselves to the Symmetric Rank One (SR1) and the Broyden update versions.

Technically, both types deal with the global solution update equation (46) and modify it specifically. Let us consider (46) written in terms of the nodal displacements

50 $$\begin{aligned} \hat{\varvec{u}}_G^n:=\hat{\varvec{u}}_G^{n-1}+(\Delta \hat{\varvec{u}}_G)^n \quad \mathrm {s.t.}\, (\Delta \hat{\varvec{u}}_G)^n=\mathsf {K}_G^{-1}\mathsf {r}_\Gamma ^n, \end{aligned}$$

where, owing to ($\hbox {G}_\mathrm {incr}$), one has

$$\begin{aligned} \mathsf {K}_G:=\int _\Omega (\varvec{B}_u^G)^\mathsf {T} \, \widetilde{\mathbb {C}} \, \varvec{B}_u^G, \end{aligned}$$

with $\widetilde{\mathbb {C}}$ as a $3\times 3$-matrix representation of $\mathbb {C}$, and

$$\begin{aligned} \mathsf {r}_\Gamma ^n:=\int _\Gamma (\varvec{N}_u^G)^\mathsf {T} \, (\varvec{\lambda }_F^n-\varvec{\lambda }_F^{n-1}) +\int _\Gamma (\varvec{N}_u^G)^\mathsf {T} \, (\varvec{\lambda }_C^n-\varvec{\lambda }_C^{n-1}). \end{aligned}$$

Aitken’s method modifies (50) at any iteration $n\ge 2$ introducing the damping factor $\omega _{n-1}=$ $f(\omega _{n-2},$ $(\Delta \hat{\varvec{u}}_G)^{n-1},$ $(\Delta \hat{\varvec{u}}_G)^n)$ s.t. $\omega _0=1$ as follows:

51 $$\begin{aligned} \hat{\varvec{u}}_G^n:=\hat{\varvec{u}}_G^{n-1}+\omega _{n-1}(\Delta \hat{\varvec{u}}_G)^n, \end{aligned}$$

whereas the Quasi-Newton correction modifies (50) at any iteration $n\ge 2$ by replacing the matrix $\mathsf {K}_G$ with $\widetilde{\mathsf {K}}^n=$ $f(\widetilde{\mathsf {K}}^{n-1},\mathsf {r}_\Gamma ^n,(\Delta \hat{\varvec{u}}_G)^{n-1})$ s.t. $\widetilde{\mathsf {K}}^1=\mathsf {K}_G$, thus resulting in

52 $$\begin{aligned} \hat{\varvec{u}}_G^n:=\hat{\varvec{u}}_G^{n-1}+(\widetilde{\mathsf {K}}^n)^{-1}\mathsf {r}_\Gamma ^n. \end{aligned}$$

In (51), we explicitly have

53 $$\begin{aligned} \omega _{n-1}:=\omega _{n-2} \frac{ ((\Delta \hat{\varvec{u}}_G)^{n-1})^\mathsf {T} \left( (\Delta \hat{\varvec{u}}_G)^{n-1} - (\Delta \hat{\varvec{u}}_G)^n \right) }{ |(\Delta \hat{\varvec{u}}_G)^{n-1} - (\Delta \hat{\varvec{u}}_G)^n|^2 }, \quad {n\ge 2}, \end{aligned}$$

with $\omega _0=1$. In (52), the SR1 update formula implies

54 $$\begin{aligned} \widetilde{\mathsf {K}}^n:=\widetilde{\mathsf {K}}^{n-1} -\frac{ \mathsf {r}_\Gamma ^n (\mathsf {r}_\Gamma ^n)^\mathsf {T} }{ (\mathsf {r}_\Gamma ^n)^\mathsf {T} (\Delta \hat{\varvec{u}}_G)^{n-1} }, \quad {n\ge 2}, \end{aligned}$$

whereas the Broyden update reads as

55 $$\begin{aligned} \widetilde{\mathsf {K}}^n:=\widetilde{\mathsf {K}}^{n-1} -\frac{ \mathsf {r}_\Gamma ^n ((\Delta \hat{\varvec{u}}_G)^{n-1})^\mathsf {T} }{((\Delta \hat{\varvec{u}}_G)^{n-1})^\mathsf {T} (\Delta \hat{\varvec{u}}_G)^{n-1} }, \quad {n\ge 2}. \end{aligned}$$

In both cases $\widetilde{\mathsf {K}}^1=\mathsf {K}_G$. Further details about computing the inverse matrices, efficient data storage etc. can be found e.g. in [6, 39]. Also, following Conn et al. [62], the SR1 update formula (54) is used only if

$$\begin{aligned} |(\mathsf {r}_\Gamma ^n)^\mathsf {T} (\Delta \hat{\varvec{u}}_G)^{n-1}|\ge c_1|\mathsf {r}_\Gamma ^n|\, |(\Delta \hat{\varvec{u}}_G)^{n-1}|, \end{aligned}$$

with a constant $c_1\in (0,1)$. Otherwise, we simply set $\widetilde{\mathsf {K}}^n:=\widetilde{\mathsf {K}}^{n-1}$. This helps preventing the convergence issue of the global/local procedure using the SR1 based relaxation.

**Fig. 12:**
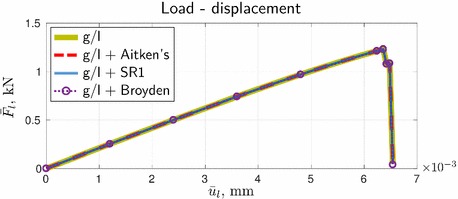
Comparison of the load-displacement curves

**Fig. 13:**
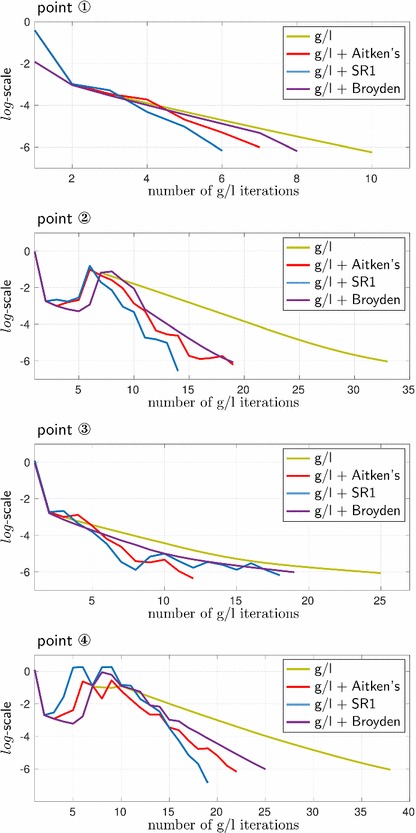
Convergence behavior of the different versions of the global/local iterative solution process at four different loading steps (the points 1–4 from Fig. 5c), illustrated in terms of the indicator $\eta$

**Fig. 14:**
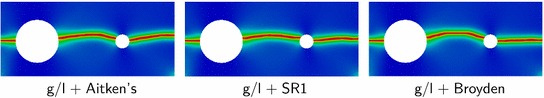
Comparison of the phase-field profiles computed with the various acceleration/relaxation versions of the global/local approach

**Fig. 15:**
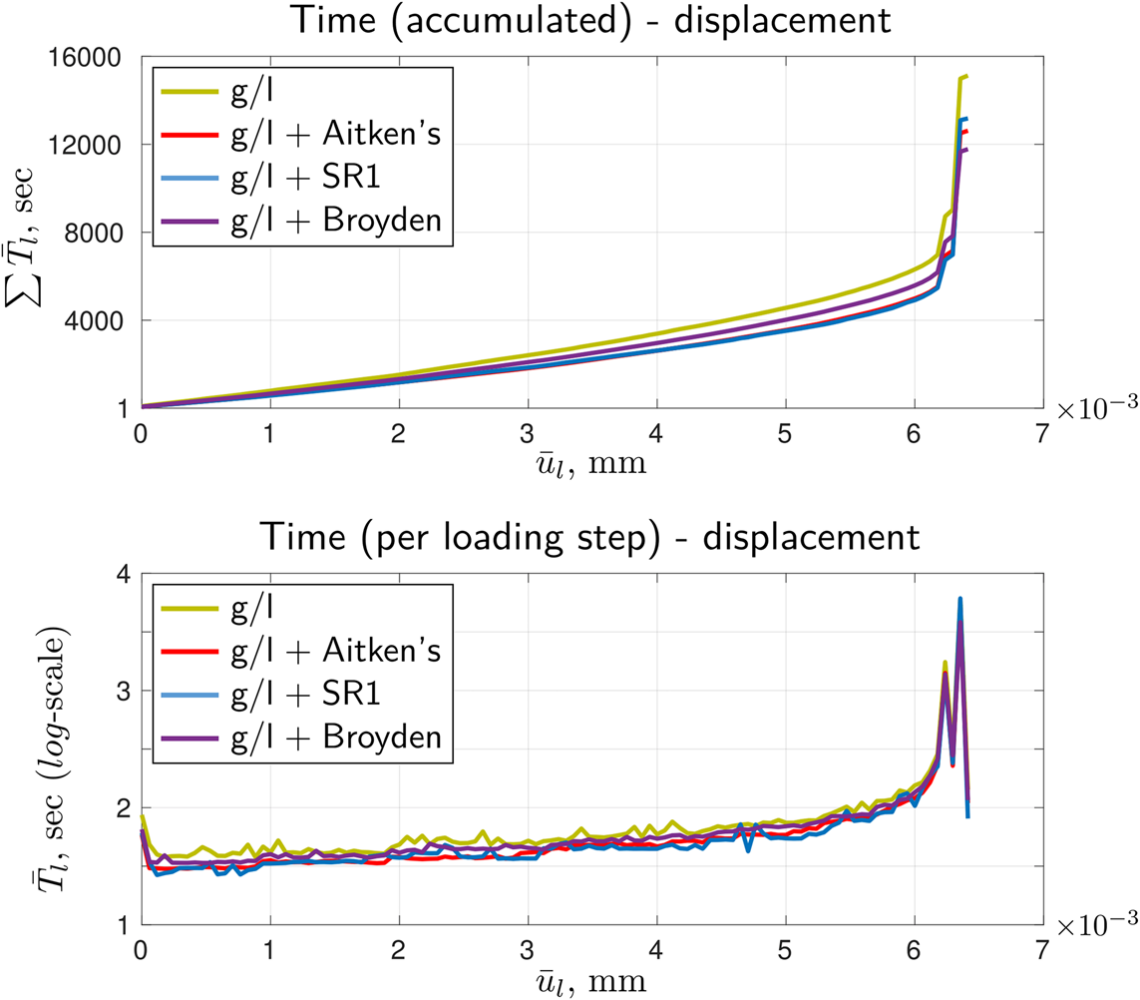
Comparison of the time-displacement curves presented in different formats

The results obtained with the relaxation/acceleration techniques are depicted in Figs. 12–15. As can be seen from Fig. 12, all three considered techniques yield identical load-displacement curves, also identical to the curve obtained from the global/local procedure with no relaxation/acceleration.

Similarly to Figs. 9 and 13 presents and compares the convergence of the global/local iterative procedure and its acceleration/relaxation versions at the four loading steps of interest. Here, we only plot the indicator $\eta$ and not its ingredients. For a given point, the amount of iterations required for the convergence of the solution process in all acceleration/relaxation techniques is similar, but is less (in some cases, significantly) than in the original unaccelerated case.

Figure 14 compares the phase-field solutions of the global/local formulations computed using the corresponding acceleration/relaxation techniques. It can be observed that even though the load-displacement curves are identical in all cases, the corresponding phase-field profiles are not. This can be explained, first of all, by the solution non-uniqueness of the original reference phase-field formulation, and, secondly, by the fact that the global/local formulation is only the approximation of the reference one.

From the time-displacement curves comparison in terms of both ‘cumulative time’ and ‘time per loading step’, Fig. 15, it can be concluded that the desired improvement of efficiency of the original procedure has indeed been achieved. However, in the global time scale, all three techniques have a very similar effect, at least for the considered example.

## Conclusions

We combined the adoption of non-intrusive global/local approaches with phase-field modeling of brittle fracture, with the main objective to pave the way for a wide adoption of this framework for industrial applications within legacy codes. We investigate the convergence performance of the fixed-point scheme used for the global/local iterations and showed that the obtained results are identical to the reference phase-field solution. In order to accelerate the observably quite slow convergence behavior, especially close to and beyond the peak point(s) of the load-displacement response, we also equipped the global/local solution update procedure with relaxation/acceleration techniques such as Aitken’s $\Delta ^2$-method, the Symmetric Rank One and Broyden’s methods. Findings showed that the iterative convergence can be improved significantly, to a similar extent for all investigated methods. Aitken’s $\Delta ^2$-method is probably the most convenient choice for the implementation of the approach within legacy codes, as this method needs only tools which are often used for the so-called sub-modeling strategy, which is well known and widely used in industrial contexts.

Several extensions and improvements of the proposed framework are foreseen, such as the study of the effect of global modeling choices on the iterative convergence behavior, the investigation of alternative boundary conditions for the coupling, the implementation of mortar-type approaches to enable non-matching meshes at the boundary between local and complementary domains. Moreover, in practical applications when i.e. the evolving localization areas are not known á priori, the global/local approach must be supplied with the possibility of the adaptive choice of the local domain $\Omega _L$. The representation result for *d*, namely,

56 $$\begin{aligned} d(\mathbf x )=\frac{1}{|V|} \int _V g(\varvec{\xi }) \frac{ \frac{2\ell }{G_c}\Psi ^+(\mathbf x +\varvec{\xi }) }{ 1+\frac{2\ell }{G_c}\Psi ^+(\mathbf x +\varvec{\xi }) } \,\mathrm {d}\varvec{\xi }, \end{aligned}$$

obtained in Ambati et al. [15], p. 392, equation (34) suggests that $\Psi ^+$, or rather the history field ${\mathcal {H}}_l$, may serve as an “indicator” for the adaptive choice of $\Omega _L$. In (56), $V\subset \Omega$ is an averaging volume, $\varvec{\xi }\in V$, and $g(\varvec{\xi })$ is a bell-shaped function, typically a Gaussian, such that $\frac{1}{|V|}\int _V g(\varvec{\xi })\,\mathrm {d}\varvec{\xi }=1$ holds. These issues are all open for further research.
